# Protein Modifications and Metabolic Alterations in the Rat Striatum Following Oil Mist Particulate Matter Exposure Revealed via Untargeted Metabolomics and Phosphoproteomics

**DOI:** 10.3390/toxics14030249

**Published:** 2026-03-12

**Authors:** Huipeng Nie, Xuan Liu, Yue Shi, Huanliang Liu, Wenqing Lai, Kang Li, Lei Tian, Zhuge Xi, Bencheng Lin

**Affiliations:** Tianjin Key Laboratory of Risk Assessment and Control Technology for Environment and Food Safety, Military Medical Sciences Academy, Academy of Military Sciences, Tianjin 300050, China

**Keywords:** metabolomics, phosphoproteomics, OMPM, striatum, DA

## Abstract

Prolonged occupational exposure to oil mist particulate matter (OMPM) poses health risks, yet its neurotoxic effects and underlying mechanisms remain poorly understood. Here, OMPM generated from turbine oil commonly used in occupational labor environments was used to expose rats. The rats were divided into the control and OMPM groups. Following 42 days of exposure, a multidimensional assessment was performed using untargeted metabolomics, phosphoproteomics, behavioral testing, hematoxylin–eosin (HE) staining, transmission electron microscopy (TEM), colorimetric assays, enzyme-linked immunosorbent assay, and Western blotting (WB) to evaluate metabolic alterations, protein phosphorylation, and tissue integrity in the striatum. Integrated omics analyses revealed that differentially phosphorylated proteins and metabolites were remarkably enriched in dopaminergic synapse, Parkinson’s disease, and amphetamine addiction pathways (FDR < 0.05), with a regulatory axis involving L-tyrosine, tyrosine hydroxylase (TH), and dopamine (DA) identified. OMPM-exposed rats exhibited depression- and anxiety-like behaviors, alongside striatal pathological and ultrastructural damage. Biochemical analyses showed elevated malondialdehyde and reactive oxygen species levels; reduced superoxide dismutase, glutathione, and glutathione peroxidase activities and total antioxidant capacity; increased glutathione disulfide and inducible nitric oxide synthase expression; and decreased DA and L-tyrosine levels. Additionally, proinflammatory mediators (IL-1β, IL-6, TNF-α, MCP-1, and PGD_2_) were significantly upregulated in the striatum. WB analysis further confirmed significant reductions in the relative phosphorylation levels of key regulators in dopaminergic and calcium signaling pathways, including CALM3, CaMK2b, GSK-3β, PRKCG, and TH. Collectively, these findings reveal critical molecular and biochemical alterations in the rat striatum following OMPM exposure and provide a mechanistic basis for understanding depression-like behaviors associated with prolonged OMPM exposure in occupational workers.

## 1. Introduction

During the manufacturing, commissioning, and maintenance of industrial equipment, various industrial lubricants and metalworking fluids are used. These fluids readily release oil-mist particulate matter (OMPM) in the occupational environment. OMPM predominantly consists of submicron-sized particles, with diameters primarily below 2.5 μm. These particles are easily inhalable and, upon prolonged exposure, pose a significant threat to human health [1]. Our previous work [2] revealed that OMPM had irregular morphology, heterogeneous distribution, and variable particle sizes and was mainly composed of aluminosilicates, inorganic salt crystals, organic carbon, and amorphous carbon. The main organic constituents include C20–C40 saturated hydrocarbons and bicyclic aromatic hydrocarbons, as well as phenolic, aniline, and other oxygen-containing compounds such as ketones, esters, and ethers. These complex physicochemical characteristics may collectively contribute to its enhanced biotoxicity. Prolonged exposure to OMPM has been shown to exert multiple adverse effects on the health of occupational workers, including skin allergies, respiratory diseases such as asthma and pulmonary inflammation, immune dysfunction, and an increased risk of lung cancer and brain cancer [3,4,5]. Building on evidence that particulate matter (PM_2.5_) exposure induced neuroinflammation and depression-like behaviors in both animal models and human cohorts [6,7], it is plausible that sustained OMPM exposure may also exert similar neurobehavioral effects in occupational workers. Despite this inference, the biological and molecular mechanisms underlying OMPM-associated neurobehavioral alterations remain largely unexplored.

The striatum is recognized as the primary input structure of the basal ganglia, where signals related to motivation, emotion, habit formation, cognition, and sensorimotor function are integrated and subsequently translated into observable behavioral outputs [8,9,10]. Dysregulated neural activity within the striatum has been implicated in the development of psychiatric disorders [11]. Striatal function is modulated by a complex network of neurotransmitters and neuromodulators, among which dopamine (DA) plays a key role [12]. DA-mediated signaling is essential for normal striatal operation and is primarily driven by long-range dopaminergic projections originating from the ventral tegmental area and substantia nigra pars compacta (SNc). These projections regulate motor control, cognition, and motivation [13]. The maintenance of striatal functional homeostasis depends critically on DA biosynthesis, which is initiated and regulated through a tightly controlled molecular network. DA synthesis begins with the transport of the aromatic amino acid L-tyrosine across the blood–brain barrier via specific amino acid transporters, ensuring sufficient precursor availability in the brain [14,15]. Within dopaminergic neurons, L-tyrosine is hydroxylated by tyrosine hydroxylase (TH) to form L-3,4-dihydroxyphenylalanine, representing the rate-limiting step in DA biosynthesis [16,17,18,19]. The activity and expression of TH therefore directly determine DA synthesis efficiency and overall output. As the key rate-limiting enzyme in this pathway, TH is regulated by multiple protein kinases. These include protein kinase A (PKA), protein kinase C (PKC), calcium/calmodulin-dependent protein kinase II (CaMKII), mitogen-activated protein kinase-activated protein kinase 2 (MAPKAPK2), extracellular signal-regulated kinases 1 and 2 (ERK1/2), mitogen- and stress-activated protein kinase 1 (MSK1), and p38-regulated/activated protein kinase (PRAK) [20,21]. Disruption of striatal dopaminergic signaling has been strongly associated with psychiatric and neurodegenerative disorders, and impaired DA transmission can lead to various neuromotor and psychiatric conditions, including Parkinson’s disease and substance addiction [8,22,23]. In Parkinson’s disease, progressive degeneration of SNc dopaminergic neurons and significant reductions in TH activity lead to severe DA depletion and consequent motor dysfunction [24,25]. Although the central role of the L-tyrosine-TH-DA pathway in DA biosynthesis is well established, the precise regulatory mechanisms underlying this pathway in striatal dopaminergic neurotransmission, as well as the direct contribution of its key molecular components to depression and other psychiatric disorders, remain incompletely understood.

Therefore, we hypothesize that OMPM exposure may be associated with depression-like behaviors in humans by disrupting striatal signaling pathways. However, the specific pathways involved (including DA-mediated signaling) and the key protein targets that may exert system-wide regulatory effects remain largely undefined. Metabolomics has emerged as a powerful analytical approach for the comprehensive quantitative profiling of metabolites within biological systems [2]. Additionally, protein phosphorylation is an important post-translational modification mechanism in which kinases transfer phosphate groups to specific amino acid residues of proteins, thereby altering their conformations and functions [26], and dysregulation of critical phosphorylation events is closely associated with disease initiation and progression [27,28]. Phosphoproteomic techniques are highly effective in deciphering complex signal transduction networks, facilitating the identification of disease-relevant molecular targets and thereby supporting broad translational and clinical applications [29,30,31]. In the present study, we integrated untargeted metabolomics and phosphoproteomics to systematically elucidate the molecular mechanisms underlying depression-like behaviors induced by OMPM exposure. Our findings aim to provide a theoretical foundation for understanding OMPM-related neurotoxicity and to provide a reference for the development of health management strategies for occupational workers.

## 2. Materials and Methods

### 2.1. Animals

Specific-pathogen-free Wistar rats (body weight: 180–220 g, half male and half female, *n* = 54) were purchased from Beijing Vital River Laboratory Animal Technology Co., Ltd. (Beijing, China), and each rat was the independent experimental unit. All animals were housed under standard laboratory conditions, including controlled temperature (24–26 °C), relative humidity (55–60%), and a 12 h light/dark cycle, with *ad libitum* access to standard chow and water. All experimental procedures were conducted in accordance with the guidelines of the Institutional Animal Care and Use Committee (IACUC) and were approved by the IACUC of the Military Medical Sciences Academy (Ethical Approval No.: IACUC of AMMS-04-2020-016).

### 2.2. OMPM Exposure Experiment

After 1 week of acclimatization under experimental conditions, rats were randomly assigned to either the control or the OMPM exposure group using a random number table (*n* = 18 per group). For the OMPM group, OMPM was generated using a liquid aerosol generator (HRH-WAG12, Beijing Huironghe Technology Co., Ltd., Beijing, China) and introduced into the exposure chamber of a whole-body animal exposure system (HRH-MNE3026, Beijing Huironghe Technology Co., Ltd.). This system was designed to simulate the air environment contaminated with OMPM found in typical occupational work environments, regulating exposure concentrations by adjusting specific aerosol generation parameters. The TSI 8530 DustTrak II aerosol monitor (TSI Inc., Shoreview, MN, USA) was used for real-time monitoring of the concentration in the exposure chamber; meanwhile, the filter membrane weighing method was employed three times a day during the experiment to detect the OMPM concentration in the exposure chamber so as to ensure the stability and accuracy of the experimental results. Rats in the OMPM group were subjected to continuous OMPM exposure for 42 consecutive days at 6.5 h per day. Rats in the control group were not exposed to OMPM and were maintained under identical housing and environmental conditions. Based on preliminary studies conducted by our research group, the exposure concentrations were set at 0 mg/m^3^ for the control group and 50 mg/m^3^ for the OMPM group [32]. Specifically, based on the occupational exposure limit of 5 mg/m^3^ for OMPM in workplace environments recommended by the American Conference of Governmental Industrial Hygienists (ACGIH), we determined the OMPM exposure concentration by incorporating a 10-fold safety factor, which conformed to the National Institute for Occupational Safety and Health (NIOSH)-defined “effective” immediately dangerous to life or health level (10 × REL/PEL) and was a standard inhalation toxicology extrapolation method officially documented by NIOSH. This factor accounted for interspecies and exposure duration differences between short-term animal models and lifelong human occupational exposure and ensured the concentration elicited measurable toxic effects while maintaining translational relevance to real-world occupational settings. After confirmation of successful model establishment, striatal tissues were carefully dissected and collected for subsequent analyses.

### 2.3. Open-Field Test

This test was conducted in a rectangular arena (70 × 70 × 30 cm, open top) under uniformly calibrated lighting conditions. Each rat was gently placed at the center of the arena, and spontaneous locomotor activity was recorded for 5 min using a video tracking system, which consisted of a high-definition industrial camera (Sony XCG-CG240C color industrial camera, manufactured by Sony Group Corporation, Tokyo, Japan; equipped with an IMX249 global shutter CMOS sensor, manufactured by Sony Semiconductor Solutions Corporation, Kumamoto, Japan) and TrackingMaster V4.10 behavioral analysis software (Zhongshi Dichuang Science and Technology Development Co., Ltd., Beijing, China).

### 2.4. Tail Suspension Test

Rats were acclimatized to the behavioral testing room for 1 h before experimentation by transferring their home cages to the room to minimize stress associated with environmental novelty. For the test, a strip of medical adhesive tape was affixed approximately 1 cm from the distal end of each rat’s tail. Rats were then suspended in an inverted position, with the tail tip positioned 30 cm above the test floor. Behavioral activity was continuously recorded for 5 min using a behavioral recording system (TrackingMaster, Zhongshi Dichuang Science and Technology Development Co., Ltd., Beijing, China; Sony XCG-CG240C). Upon completion of the test, the tape was carefully removed, and the rats were gently returned to their home cages.

### 2.5. Forced Swimming Test

Rats were placed in a circular transparent water tank from which they could not escape, and the water temperature was maintained at about 25 °C. Each rat was tested for 5 min, and core behavioral indicators such as immobility time were observed and recorded simultaneously using a behavioral recording system (TrackingMaster, Zhongshi Dichuang Science and Technology Development Co., Ltd., Beijing, China; Sony XCG-CG240C). After each trial, the water was completely replaced, and the cylinder was wiped with 75% ethanol to eliminate odor cues.

### 2.6. Sucrose Preference Test

The total volume of liquid in the water bottle was configured to 500 mL. During the pre-experiment, each animal was given one bottle of 1% sucrose water and one bottle of pure water at the same time, and the horizontal positions of the bottles were changed every 12 h. After the pre-experiment, the animals were fasted and deprived of water for 24 h. After 1 h of the formal experiment, the consumption of sucrose water and pure water was recorded, and the sucrose preference was calculated: sucrose preference (%) = sucrose water consumption/(sucrose water consumption + pure water consumption) × 100%. During the test, interference from personnel, noise, and obvious environmental changes should be avoided.

### 2.7. Kyoto Encyclopedia of Genes and Genomes (KEGG) Pathway Analysis

Differentially expressed proteins, differentially modified peptides, genes, and metabolites, identified by high-throughput phosphoproteomics and metabolomics assays in OMPM-exposed and control samples with statistical significance, were annotated and mapped to KEGG pathways using the KEGG database (http://www.kegg.jp/, accessed on 1 January 2026), and non-specific annotation results were filtered out to ensure annotation reliability. Pathway enrichment analysis was then conducted via a hypergeometric test, with FDR < 0.05 set as the threshold to screen significantly affected biological pathways for both phosphoproteomic and metabolomic datasets. KEGG annotation and enrichment results derived from the two omics datasets were integrated using R software (version 3.5.1), and Venn diagrams and bar plots were generated for data visualization (the top 10 co-annotated pathways ranked by the number of differential molecules were presented as bar plots).

### 2.8. Expression Profiles of Differentially Modified Peptides in Shared Significantly Enriched KEGG Pathways

Quantitative data for the target set of differentially modified peptides were normalized, with expression values scaled to the range of −1 to 1. Subsequently, two-dimensional hierarchical clustering was performed using R software (version 3.5.1) based on both sample grouping and peptide expression levels. Euclidean distance was applied as the distance metric, and complete linkage was used as the clustering method. Heatmaps were then generated to visualize the expression profiles of differentially modified peptides across shared significantly enriched KEGG pathways.

### 2.9. Correlation Analysis

Quantitative data for all significantly differentially expressed genes, proteins, and modified peptides were first normalized using log_2_ transformation. Similarly, quantitative data for significantly differentially expressed metabolites and lipids were normalized by log_2_ transformation. Pearson correlation coefficients were then calculated to assess the relationships among these variables. The R package corrplot (R software, version 3.5.1) was used to construct the correlation matrix heatmaps and generate the corresponding correlation files.

### 2.10. Hematoxylin–Eosin (HE) Staining

Fixed rat striatal tissues were sequentially subjected to graded ethanol dehydration, xylene clearing, and paraffin embedding. Serial sections (thickness = 4 μm) were prepared and subsequently deparaffinized in xylene. The sections were then stained with the HE procedure following standard protocols, dehydrated through a graded ethanol series, and mounted with neutral balsam. Histopathological morphological changes were examined under a light microscope, and representative micrographs were captured for documentation.

### 2.11. Transmission Electron Microscopy (TEM)

Rat striatal tissues were fixed in 2.5% glutaraldehyde for 4 h and post-fixed in 1% osmium tetroxide for 2 h. The samples were then dehydrated through a graded ethanol series, replaced with acetone, and embedded in epoxy resin. Ultrathin sections (70 nm) were prepared and subjected to double staining with uranyl acetate and lead citrate. The sections were examined using a transmission electron microscope (HT7700; Hitachi, Tokyo, Japan), and images were captured for ultrastructural analysis.

### 2.12. Enzyme-Linked Immunosorbent Assay (ELISA)

Rat striatal tissues were homogenized to prepare tissue lysates. ELISAs were performed strictly according to the manufacturers’ instructions of the commercial kits from Beyotime Biotechnology (Shanghai, China) to quantify DA, interleukin-1β (IL-1β), interleukin-6 (IL-6), tumor necrosis factor-α (TNF-α), monocyte chemoattractant protein-1 (MCP-1), and prostaglandin D_2_ (PGD_2_) levels in striatal tissues. Standard curves were generated using the optical density values and known concentrations of the provided standards, and analyte concentrations in the samples were calculated from the corresponding standard curve equations.

### 2.13. Colorimetric Assay

Rat striatal tissues were collected and homogenized thoroughly in pre-chilled normal saline at a ratio of 1:9 (*w*/*v*) on an ice bath, followed by centrifugation at 12,000× *g* for 15 min at 4 °C. The supernatant was harvested and subjected to subsequent detections. In strict accordance with the manufacturer’s instructions of the corresponding commercial kits, the contents of malondialdehyde (MDA), reactive oxygen species (ROS), reduced glutathione (GSH), oxidized glutathione (GSSG), and L-tyrosine, as well as the activities of superoxide dismutase (SOD), glutathione peroxidase (GSH-Px), total antioxidant capacity (T-AOC), and inducible nitric oxide synthase (iNOS) in the supernatant, were determined.

### 2.14. Western Blotting (WB)

Rat striatal tissues were homogenized and lysed in radioimmunoprecipitation assay buffer, followed by centrifugation at 12,000 r/min for 10 min at 4 °C to collect the supernatants. Protein concentrations were determined using the bicinchoninic acid assay. Equal amounts of protein were separated via SDS-PAGE and transferred onto polyvinylidene difluoride membranes. The membranes were blocked with nonfat milk and incubated overnight at 4 °C with primary antibodies against p-CALM3 (1:500, Affinity, Cincinnati, OH, USA), p-CaMK2b (1:500, Affinity, Cincinnati, OH, USA), p-GSK-3β (1:1000, Proteintech, Rosemont, IL, USA), p-PRKCG (1:500, Affinity, Cincinnati, OH, USA), p-TH (1:500, Affinity, Cincinnati, OH, USA), CALM3 (1:1000, Proteintech, Rosemont, IL, USA), CaMK2b (1:1000, Proteintech, Rosemont, IL, USA), GSK-3β (1:1000, Proteintech, Rosemont, IL, USA), PRKCG (1:1000, Proteintech, Rosemont, IL, USA), and TH (1:1000, Proteintech, Rosemont, IL, USA). After washing, membranes were incubated with HRP-conjugated goat anti-rabbit IgG and HRP-conjugated goat anti-mouse IgG secondary antibodies (1:4000, Proteintech, Rosemont, IL, USA) for 1 h at 4 °C. Immunoreactive bands were visualized using an enhanced chemiluminescence detection kit. Glyceraldehyde-3-phosphate dehydrogenase (1:2000, Proteintech, Rosemont, IL, USA) was used as the internal loading control. Images were acquired using a Tanon 5200 Automatic Chemiluminescence Imaging System (Tanon, Shanghai, China), and band intensities were quantified using Gel-Pro Analyzer 32 software.

### 2.15. Statistical Analysis

GraphPad Prism 10.1 software (GraphPad Software, San Diego, CA, USA) and SPSS software version 25 (SPSS Inc., Chicago, IL, USA) were employed for data visualization and statistical analysis. All data are expressed as the mean ± standard deviation. Intergroup comparisons were performed using an independent-sample *t*-test and a non-parametric test (Mann–Whitney U test). MVDA was performed on differentially expressed abundant proteins, modified peptides, genes, and metabolites with SIMCA Version 14.1. A value of *p* < 0.05 was considered statistically significant.

## 3. Results

### 3.1. Common Metabolic Pathways Involving Phosphorylated Proteins and Metabolites

**Figure 1:**
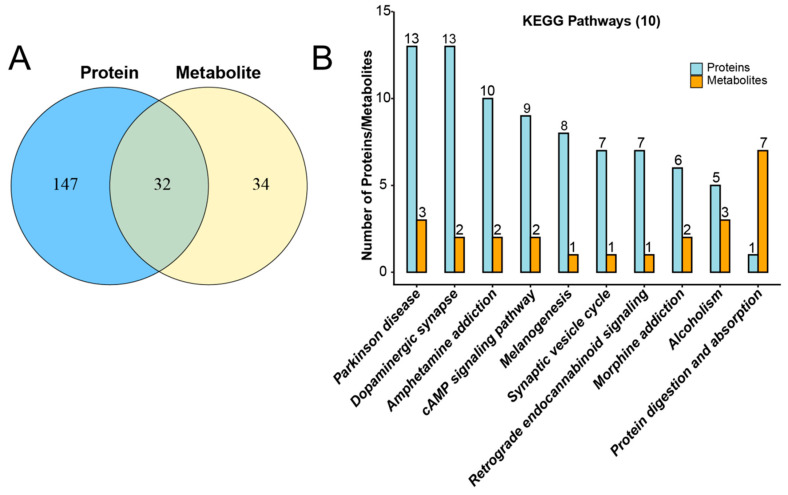
Common metabolic pathways involving phosphorylated proteins and metabolites were analyzed using R software, version 3.5.1: (**A**) The blue circle represents the phosphoproteome, and the number within the circle indicates the total number of pathways annotated by all differentially modified proteins. The yellow circle represents the metabolome, and the number within the circle indicates the total number of pathways annotated by all differentially expressed metabolites. The number in the overlapping region of the blue and yellow circles denotes the pathways shared between the two omics datasets. (**B**) The abscissa shows the names of KEGG pathways annotated by the identified differentially modified proteins or metabolites, and the ordinate indicates the number of modified proteins or metabolites annotated in each pathway. Blue bars represent the phosphoproteome, with numbers above the bars indicating the number of modified proteins identified in each pathway. Orange bars represent the metabolome, with numbers above the bars indicating the number of metabolites identified in each pathway. Paired blue and orange bars correspond to the same pathway and are arranged from left to right according to the total number of annotated modified proteins and metabolites.

Alterations in protein phosphorylation are associated with signal pathway remodeling, which may be linked to metabolic perturbations [33]. To identify common metabolic pathways that may involve coordinated changes in phosphorylated proteins and metabolites, we performed a comparative analysis based on KEGG pathway annotation. Specifically, KEGG pathways enriched in differentially modified proteins and differentially expressed metabolites in the OMPM and control groups were statistically summarized, and the number of pathways shared between the two omics datasets was visualized using a Venn diagram. As shown in Figure 1A, 147 pathways were identified in the phosphoproteomics dataset and 34 pathways in the metabolomics dataset, with 32 pathways overlapping between the two omics layers. These results demonstrate a strong overlap in the KEGG-annotated pathways associated with differentially modified proteins and differentially expressed metabolites between the OMPM and control groups, suggesting a potential coordination between protein phosphorylation changes and metabolic alterations in response to OMPM exposure. To further characterize these common metabolic pathways, the top 10 KEGG signaling pathways with the highest numbers of co-annotated phosphorylated proteins and metabolites were selected and are presented in a histogram. Additionally, the number of differentially modified proteins and differentially expressed metabolites annotated in the major shared pathways was determined. As shown in Figure 1B, a comparison of the OMPM and control group indicated that the pathways showing the most prominent co-alterations in both modified proteins and metabolites were Parkinson’s disease, dopaminergic synapse, and protein digestion and absorption. Overall, the number of differentially modified proteins in most pathways between the OMPM and control groups was substantially higher than that of metabolites, indicating that protein phosphorylation changes are the predominant molecular feature associated with the pathway alterations observed in this study. Nevertheless, certain pathways also exhibited distinct metabolite-level alterations, highlighting the concurrent changes in both phosphoproteomic and metabolic layers in response to OMPM exposure.

### 3.2. Highly Active Common Metabolic Pathways Across the Two Omics Layers

**Figure 2:**
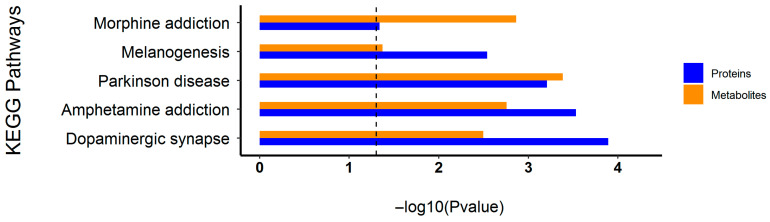
Highly active common metabolic pathways across the two omics layers. The abscissa represents pathway enrichment significance, expressed as the log-transformed *p* value, whereas the ordinate indicates the names of KEGG pathways. Each bar corresponds to one pathway; blue bars represent the phosphoproteome, and orange bars represent the metabolome. The vertical dashed line represent the significance threshold.

The enrichment significance of differentially phosphorylated proteins and metabolites within each KEGG signaling pathway was assessed to identify pathways with concomitant molecular alterations at both the phosphoproteomic and metabolomic levels. These pathways were characterized by a marked increase in the number of differentially phosphorylated proteins (showing both hyperphosphorylation and hypophosphorylation) and altered metabolites, as well as consistent detection of these molecules across samples. As shown in Figure 2, the pathways with consistent and robust molecular changes identified in the OMPM vs. control comparison included the dopaminergic synapse, the KEGG-annotated Parkinson’s disease, and amphetamine addiction. In these pathways, the enrichment significance of both differentially phosphorylated proteins and metabolites exceeded the defined threshold, with differential proteins showing both increased and decreased phosphorylation levels, demonstrating a strong overlap of differential molecules across the two omics layers and identifying these pathways as key molecular feature pathways linked to OMPM exposure. The consistent molecular alterations observed in these pathways suggest that they may be closely associated with striatal molecular changes in response to OMPM exposure.

### 3.3. Expression Characteristics of Differentially Modified Peptides in Common Metabolic Pathways

**Figure 3:**
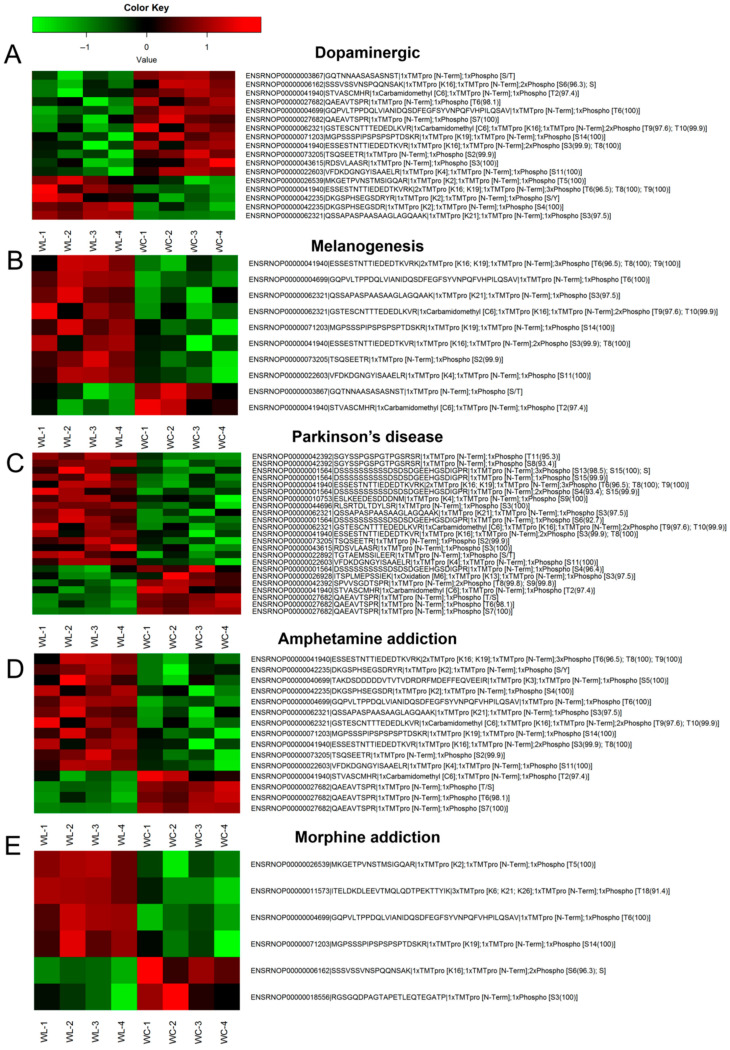
Expression characteristics of differentially modified peptides in common pathways: (**A**–**E**) The abscissa represents biological samples, divided into two groups: the OMPM group (WL) and the control group (*n* = 4 per group). Each row on the right ordinate corresponds to one modified peptide, named as follows: Ensembl ID of the protein corresponding to the differentially modified peptide + amino acid sequence of the peptide + modification type. Color coding indicates normalized expression changes in the modified peptides, with red and green representing upregulated and downregulated expression, respectively.

To intuitively illustrate the phosphorylation characteristics of modified peptides within KEGG signaling pathways that were significantly enriched in both the phosphoproteome and metabolome, differentially modified peptides from these pathways were compiled, and their patterns of upregulation and downregulation between groups were analyzed using heatmaps. Figure 3A illustrates the intergroup phosphorylation differences in differentially modified peptides in the dopaminergic synapse (rno04728) pathway. Proteins exhibiting significantly differentially modified peptides in the OMPM vs. control comparison included glycogen synthase kinase 3 beta (GSK-3β, ENSRNOP00000003867), calcium voltage-gated channel subunit alpha1 B (CACNA1B, ENSRNOP00000006162), CaMKII alpha (CAMK2A, ENSRNOP00000041940), TH (ENSRNOP00000027682), PKC alpha (PRKCA, ENSRNOP00000004699), CaMKII gamma (CAMK2G, ENSRNOP00000062321), PKC gamma (PRKCG, ENSRNOP00000071203), CaMKII beta (CaMK2b, ENSRNOP00000073205), inositol 1,4,5-trisphosphate receptor type 2 (ITPR2, ENSRNOP00000043615), calmodulin 3 (CALM3, ENSRNOP00000022603), G protein subunit gamma 3 (GNG3, ENSRNOP00000026539), and glutamate ionotropic N-methyl-D-aspartate receptor (NMDAR) subunit 2A (GRIN2A, ENSRNOP00000042235). Figure 3B–E show the intergroup phosphorylation differences in differentially modified peptides in the melanogenesis (rno04916), Parkinson’s disease (rno05012), amphetamine addiction (rno05031), and morphine addiction (rno05032) pathways, respectively. Specifically, the rno04916 pathway involved differential proteins including CAMK2A/G/B, PRKCA/G, CALM3, and GSK-3β. The rno05012 pathway included microtubule-associated protein tau (MAPT), NADH: ubiquinone oxidoreductase core subunit V3 (NDUFV3), proteasome subunit alpha 3/5 (PSMA3/5), and ubiquinol-cytochrome c reductase core protein 1 (UQCRC1), among others. The rno05031 pathway involved CAMK2A/G/B, GRIN2A, syntaxin 1A (STX1A), PRKCG, and TH, whereas the rno05032 pathway contained GNG3, gamma-aminobutyric acid type B receptor subunit 2 (GABBR2), PRKCA/G, CACNA1B, and phosphodiesterase 7B (PDE7B). Overall, both hyperphosphorylated and hypophosphorylated peptides were significantly enriched in pathways, including the dopaminergic synapse.

These findings suggest that OMPM exposure is associated with altered phosphorylation states of key neuronal kinases in signaling pathways closely linked to neurotransmission and synaptic plasticity, with these molecular changes correlated with molecular perturbations in the central nervous system observed in this study. Collectively, this study provides preliminary insights into key phosphorylated proteins and associated signaling pathways linked to OMPM exposure, which represents preliminary experimental evidence for further investigating the potential molecular correlates underlying OMPM exposure-induced depression-like behaviors.

### 3.4. Expression Regulation Between Phosphorylated Proteins and Metabolites

**Figure 4:**
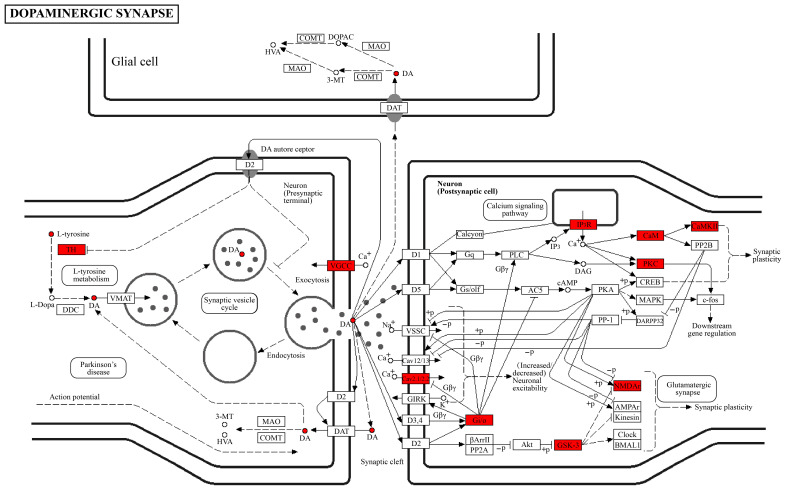
Pathway diagram of differentially phosphorylated proteins and differential metabolites. Boxes represent modified proteins, and circles represent metabolites. Red indicates significantly differentially phosphorylated proteins and metabolites.

To explore the potential associations between phosphorylated proteins and metabolites, proteins corresponding to differentially modified peptides and differential metabolites were mapped onto the same KEGG pathways. This approach enabled intuitive visualization of expression patterns and co-occurring differences between modified proteins and metabolites within a single pathway context. As shown in Figure 4, the analysis focused on the putative core DA synthesis pathway. In this pathway, L-tyrosine is known as the precursor substrate for DA synthesis, while TH, the rate-limiting enzyme, is widely recognized to be associated with DA production efficiency. DA has been characterized as a key neurotransmitter linked to neuronal signaling. In addition to these core components, significant expression changes were observed in multiple regulatory molecules, including voltage-gated calcium channels (e.g., CaV2.1/2.2), G protein inhibitory/olfactory subunits (Gi/o), GSK-3, NMDARs, inositol 1,4,5-trisphosphate receptors (IP3R), calmodulin (CaM), PKC, and CaMKII. These molecules were associated with a variety of distinct but interconnected biological processes. Specifically, CaV2.1/2.2 mediate calcium influx to facilitate vesicular exocytosis and DA release; Gi/o proteins function downstream of DA receptors and are involved in dopaminergic signal transduction; NMDARs, CaM/CaMKII, and PKC show known associations with synaptic strength and plasticity; and GSK-3 and IP3R are implicated in calcium signaling and cell survival pathways, which are linked to cellular homeostasis. Collectively, these differentially modified molecules exhibit coordinated changes with DA synthesis, release, and signal transmission-related processes following OMPM exposure, suggesting their potential involvement in these biological processes. Many of these molecules are also shared with the KEGG-annotated Parkinson’s disease pathway. The observed enrichment likely reflects the shared abundance of synaptic proteins among these pathways, rather than specific pathological changes associated with Parkinson’s disease. Integrated analysis of these components provided a systematic description of the molecular patterns within the dopaminergic system and revealed preliminary molecular associations, which may represent a foundation for further investigating the potential molecular correlates of depression-like behaviors.

### 3.5. Correlation of Metabolic Pathways

**Figure 5:**
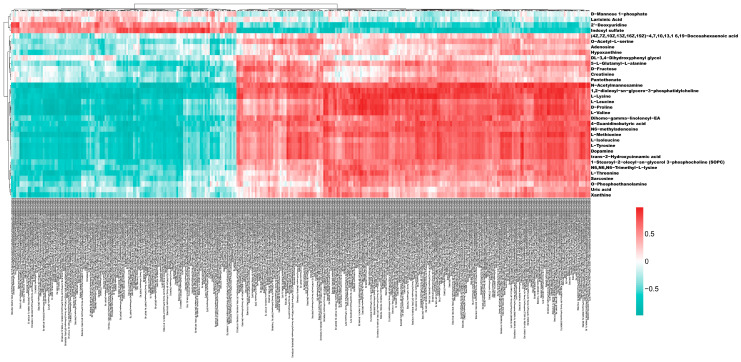
Hierarchical clustering heatmap was used for correlation analysis: each row represents a significantly differential metabolite, and each column shows a significantly differential modified peptide. The dendrogram on the left shows the clustering results of differential metabolites, while the one on the top illustrates those of differential modified peptides. Significantly different metabolites or modified peptides in the same cluster shared similar expression patterns. Each cell in the heatmap denotes the correlation coefficient (r), which was color-coded: red for positive correlation (r > 0) and green for negative correlation (r < 0), with deeper colors indicating stronger correlations.

Interactions among metabolic pathways are often mediated by key hub targets, and alterations in these hubs can propagate changes throughout the entire metabolic network. To further explore the potential associations between differentially modified proteins and differential metabolites, Pearson correlation analysis was performed to calculate correlation coefficients (r) between significantly differentially modified peptides and significantly differential metabolites. The Pearson correlation coefficient ranges from −1 to +1, where r > 0 indicates a positive correlation and r < 0 indicates a negative correlation. As shown in Figure 5, a correlation heatmap comprising 381 elements, including 347 significantly differentially modified peptides (details shown in Table S1) and 34 significantly differential metabolites in the OMPM vs. control comparison, was generated. The heatmap revealed clear clustering of these elements into four core modules. The top-left and bottom-right modules were predominantly red (r approaching +1), indicating strong positive correlations between the corresponding modified peptides and metabolites. Conversely, the top-right and bottom-left modules were predominantly green (r approaching −1), indicating strong negative correlations between these elements. These correlation patterns reflected co-occurring molecular changes within shared biological processes, without implying direct regulatory relationships between individual molecules. Overall, the observed correlation structure demonstrated that OMPM exposure was associated with coordinated alterations in phosphorylated proteins and metabolites, which were clustered into key molecular modules. These coordinated molecular changes were correlated with the molecular perturbations observed in brain tissue signaling pathways.

### 3.6. OMPM Exposure-Induced Striatal Injury in Rats

**Figure 6:**
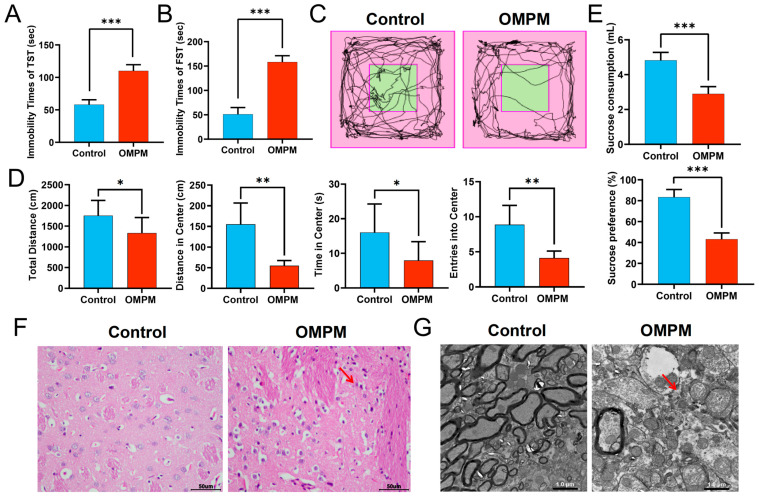
OMPM exposure induced pathological and ultrastructural damage in rat striatal neurons, accompanied by depression-like behavioral alterations: (**A**) The results of the tail suspension test. (**B**) The results of the forced swimming test. (**C**) Representative activity trajectories of rats in the control and OMPM groups during the open-field test. (Green: central zone; Pink: peripheral zone; Black lines: animal movement trajectories). (**D**) The results of the open-field test. (**E**) The results of the sucrose preference test. (**F**) HE staining of striatal tissues (scale bar = 50 μm); red arrow: nuclear pyknosis was observed in neurons of the rat striatum. (**G**) TEM images showing ultrastructural alterations in striatal neurons (scale bar = 1.0 μm); red arrow: mitochondrial swelling, membrane rupture, and cristae dissolution and fragmentation were observed in rat striatal neurons. * *p* < 0.05, ** *p* < 0.01, and *** *p* < 0.001.

**Figure 7:**
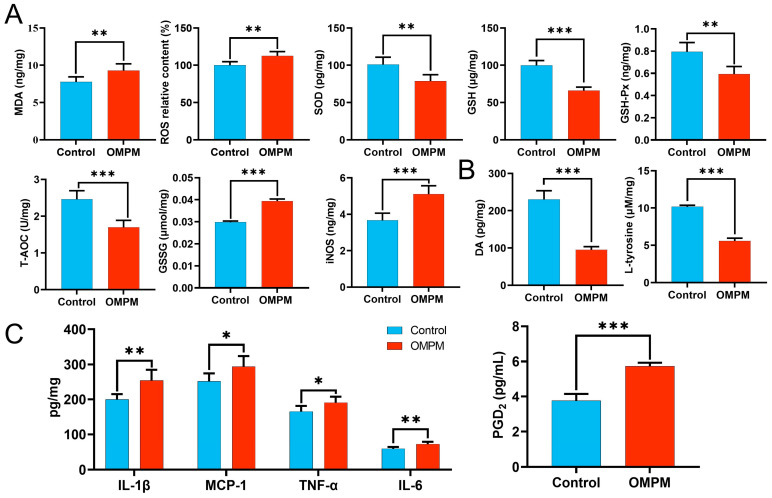
OMPM exposure induced alterations in oxidative stress-, inflammatory-, and neuro-related biomarkers in the rat striatum: (**A**) Levels of oxidative stress-related indicators (MDA, ROS, SOD, GSH, GSH-Px, T-AOC, GSSG, and iNOS). (**B**) Levels of DA and L-tyrosine. (**C**) Levels of inflammatory factors (IL-1β, IL-6, TNF-α, MCP-1, and PGD_2_). * *p* < 0.05, ** *p* < 0.01, and *** *p* < 0.001.

To elucidate the effects of OMPM exposure on behavioral phenotypes and brain tissue morphology, a tail suspension test, forced swimming test, open-field test, and sucrose preference test were conducted to assess depression-like behaviors in rats. Additionally, HE staining and TEM were performed to examine pathological and ultrastructural changes in the striatum, and oxidative stress- and inflammation-related indicators were measured in striatal tissues. Behavioral assessments revealed that rats in the OMPM group exhibited significant depression-like behaviors compared with those in the control group. Specifically, immobility time was significantly prolonged in the tail suspension test and forced swimming test (Figure 6A,B). Furthermore, open-field trajectory plots demonstrated that rats in the OMPM group displayed predominantly peripheral movement, with markedly reduced exploration of the central area (Figure 6C). Spontaneous locomotor activity was reduced in the open-field test, as evidenced by a shortened total travel distance and significant decreases in both residence time and entry frequency in the central area (Figure 6D). The rats in the OMPM group exhibited remarkable reductions in both sucrose water consumption and sucrose preference rate (Figure 6E). HE staining showed that striatal neurons in the control group were densely and orderly arranged, whereas those in the OMPM group exhibited pronounced karyopyknosis, as indicated by red arrows (Figure 6F). TEM further revealed severe ultrastructural damage in striatal neurons following OMPM exposure, including mitochondrial swelling, mitochondrial membrane rupture, and cristae dissolution and fragmentation (indicated by red arrows; Figure 6G). Biochemical analyses demonstrated that MDA and ROS levels were significantly elevated in the striatum of OMPM-exposed rats. Conversely, levels of antioxidant defenses, including SOD, GSH, GSH-Px, and T-AOC, were markedly reduced. Meanwhile, GSSG content and iNOS expression were significantly increased, whereas DA and L-tyrosine levels showed significant reductions (Figure 7A,B). Furthermore, inflammatory mediators, including IL-1β, IL-6, TNF-α, MCP-1, and PGD_2_, were significantly upregulated in striatal tissues (Figure 7C). Collectively, these results indicated that OMPM exposure induced pronounced ultrastructural damage to striatal neurons and simultaneously triggered oxidative stress imbalance and inflammatory activation in the striatum. These observations also reflect altered dopaminergic metabolism, as evidenced by the reduced levels of DA and L-tyrosine. These findings suggest that oxidative stress and inflammatory cascade responses are likely involved in OMPM-induced striatal neuronal injury.

### 3.7. Analysis of the Expression of Key Phosphorylated Proteins in the Striatum After OMPM Exposure

**Figure 8:**
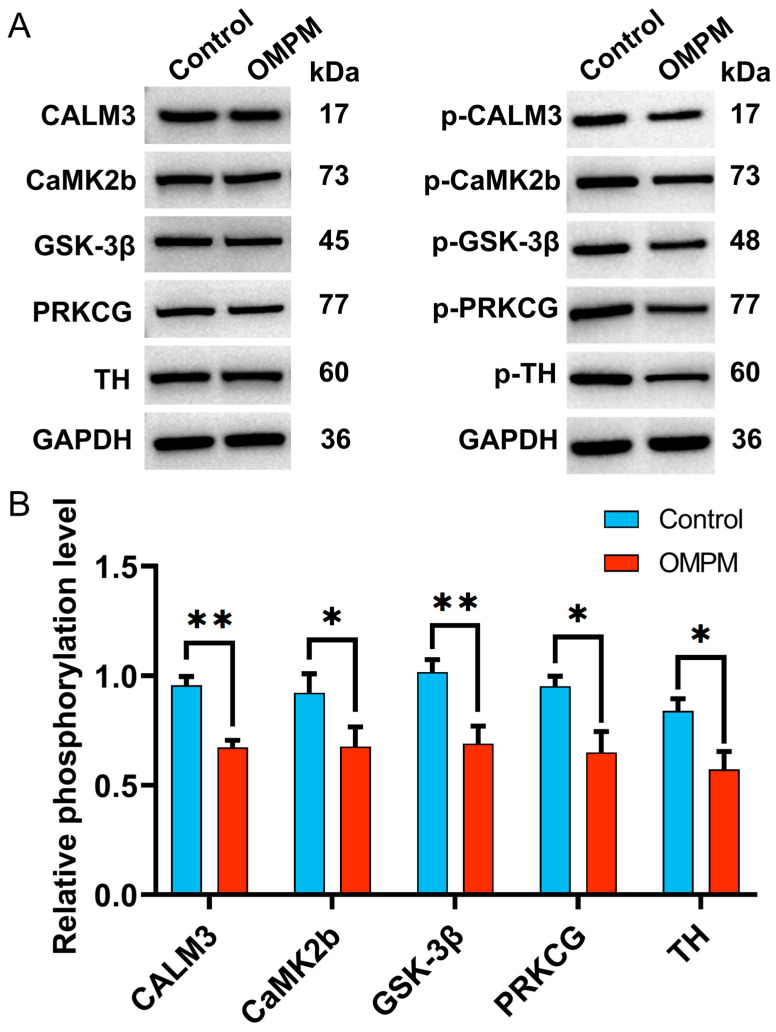
Validation and analysis of key phosphorylated protein levels: (**A**) Representative WB images of total and phosphorylated forms of CALM3, CaMK2b, GSK-3β, PRKCG, and TH in rat striatal tissues. (**B**) Quantitative analysis of band intensities shown in (**A**); relative phosphorylation level = (phosphorylated protein/GAPDH)/(total protein/GAPDH). * *p* < 0.05 and ** *p* < 0.01.

To validate the changes in the expression of differential molecules identified via untargeted metabolomics and phosphoproteomics following OMPM exposure, WB analyses were performed to detect key phosphorylated target proteins in rat striatal tissues. Compared with the control group, the relative phosphorylation levels of CALM3, CaMK2b, GSK-3β, PRKCG, and TH were significantly decreased in the striatum of rats in the OMPM group (Figure 8A,B). These phosphorylated proteins were closely associated with striatal neuronal function. Together, these findings further suggested that OMPM exposure might disrupt striatal physiological functions by affecting the phosphorylation status of proteins involved in the DA synthesis and calcium signaling pathways, which in turn supported the reliability of the omics-based screening results.

## 4. Discussion

OMPM consists of fine oil-based particles suspended in ambient air and represents a major occupational risk factor affecting the health of occupational workers. Chronic exposure to OMPM has been associated with a broad spectrum of diseases, with the respiratory system (particularly the lungs) being most severely affected. Previous studies have shown that exposure to OMPM induces pathological lung injury in rats, accompanied by significant disruption of multiple metabolic pathways [2]. Furthermore, OMPM exposure can promote hyperlipidemia-related phenotypes [34] and contribute to other adverse health effects, such as myocardial damage [1]. Although the health risks posed by OMPM are increasingly recognized, its effects on the nervous system and the underlying mechanisms remain unclear. Therefore, this study aimed to investigate OMPM-induced neurological damage and its potential mechanistic basis. Using untargeted metabolomics and phosphoproteomics, we conducted an exploratory analysis to systematically screen and decipher the molecular mechanisms underlying OMPM neurotoxicity, identifying changes in post-translational modifications and metabolites. These efforts provide initial mechanistic hypotheses and clues linking OMPM exposure to neurobehavioral abnormalities.

The striatum is a major brain structure that plays a central role in motor control, behavioral selection, and cognitive function [35,36]. To elucidate the effects of OMPM exposure on the striatum, we adopted an integrated multi-omics and experimental approach in a rat model. Untargeted metabolomics and phosphoproteomics analyses revealed that differentially phosphorylated proteins and differential metabolites exhibited consistent and stable alterations, with significant enrichment in the dopaminergic synapse pathways. Notably, within the dopaminergic synapse pathway, multiple differentially phosphorylated proteins (e.g., ITPR2, CALM3, GNG3, GRIN2A, GSK-3β, CACNA1B, TH, members of the CAMK2 family, and the PRKC family) exhibited significant intergroup differences, as confirmed via heatmap analyses. Focused investigation of the L-tyrosine-TH-DA core biosynthetic pathway further revealed that several regulatory molecules, including CaV2.1/2.2, Gi/o proteins, GSK-3, and NMDARs, may participate in the regulation of DA synthesis, release, signal transduction, and synaptic plasticity. Correlation analyses additionally demonstrated that differentially modified peptides and metabolites were divided into distinct correlation modules, with subsets of molecules exhibiting coordinated variation patterns. Behavioral assessments provided functional validation of these molecular alterations, and the findings collectively suggested that OMPM exposure was associated with significant impairment of emotion-related behaviors in rats. Further experimental validation demonstrated that OMPM exposure induced pronounced striatal structural damage, including nuclear pyknosis, disorganized cellular arrangement, and severe mitochondrial abnormalities, with concomitant significant disruption of striatal redox homeostasis and the onset of inflammatory responses. OMPM exposure has been reported to promote hyperlipidemia-associated inflammatory responses and macrophage infiltration, and accumulating evidence suggests that hyperlipidemia itself is closely linked to mitochondrial dysfunction [37,38]. Additionally, research suggests that multiple constituents of OMPM contribute to its adverse health effects [32]. Previous studies have identified that OMPM contains heavy metals and carbonyl compounds, with differences in the types and concentrations of accumulated heavy metals targeting mechanisms associated with oxidative stress (ROS/HO-1), inflammatory pathways (ROCK1/NF-κB/IL-6), and epithelial barrier damage (ZO-2/A1AT) [3], and the adverse effects of ambient particulate matter are often accompanied by oxidative stress and calcium signaling disorders [39]. OMPM exposure led to a significant reduction in DA and L-tyrosine levels and altered phosphorylation of key proteins (CALM3, CaMK2b, GSK-3β, PRKCG, and TH), as confirmed by WB, collectively suggesting that OMPM may contribute to central nervous system structural and functional abnormalities, potentially via dysregulated dopaminergic and calcium signaling pathways.

Based on our team’s previous studies on OMPM-induced rat lung and myocardial injuries, this study used untargeted metabolomics and phosphoproteomics to investigate striatal neurotoxicity and its mechanisms, achieving innovation from somatic to neuropsychiatric injury research: (1) It investigated OMPM-induced neurotoxicity in the central striatum, which helped to fill the research gap in OMPM-mediated central nervous system injury and its potential underlying mechanisms. (2) It revealed that OMPM was associated with dopaminergic synaptic disorder, synergistic oxidative stress–inflammatory injury, and anxiety and depression-like behaviors in the striatum, with potential toxic mechanisms involving the imbalance of the L-tyrosine–TH–DA axis and abnormal phosphorylation of calcium signaling and dopaminergic-related proteins. (3) It provided preliminary mechanistic support for neuropsychiatric abnormalities associated with long-term occupational OMPM exposure through multi-omics analysis integrated with behavioral and morphological assessments.

Despite these strengths, certain limitations should be acknowledged. Firstly, the relatively limited sample size may affect statistical power and restrict the generalizability of the conclusions. Secondly, the current behavioral tests may not comprehensively assess the behavioral phenotypes. Thirdly, the present study was based on an experimental animal model, which differs from real-world occupational exposure scenarios in terms of exposure dose, route, duration, and physiological metabolism. Caution is required when extrapolating these findings to occupationally exposed workers. In addition, no direct functional validation experiments were performed. Future research should focus on the following directions: conducting systematic in vivo and in vitro functional validation experiments, investigating the role of hormonal effects in nervous system injury, clarifying the dose–time–effect relationship through multi-concentration and multi-time-point exposure experiments to fully elucidate the dynamic characteristics of toxicological effects, and conducting translational studies to bridge laboratory findings with human occupational exposure scenarios. This approach will provide more robust scientific evidence to facilitate clinical translation.

## 5. Conclusions

This work focused on OMPM and employed an integrated multi-omics and multimodal analytical strategy to investigate its effects on the rat striatum. In the OMPM group, omics analyses revealed significant enrichment of pathways associated with dopaminergic synaptic signaling, accompanied by coordinated dysregulation of key proteins and metabolites and functional disturbances in dopaminergic metabolic and calcium signaling pathways. OMPM exposure was associated with marked pathological damage in the striatum, and alterations in core molecular targets were further validated via ELISA and WB analyses. Collectively, these findings provided insights into potential associative molecular alterations underlying OMPM-induced striatal injury and depression-like behaviors, offering preliminary scientific evidence to support the development of prevention and intervention strategies. Nevertheless, the detailed regulatory mechanisms require further investigation in future work.

## Data Availability

The data presented in this study are available on request from the corresponding author.

## Supplementary Materials

The following supporting information can be downloaded at: https://www.mdpi.com/article/10.3390/toxics14030249/s1, Table S1: List of significantly differential modified peptides in the hierarchical clustering heatmap (from left to right).

## Abbreviations

The following abbreviations are used in this manuscript:

CACNA1B	Calcium voltage-gated channel subunit alpha1 B
CALM3	Calmodulin 3
CaM	Calmodulin
CaMKII	Calcium/calmodulin-dependent protein kinase II
DA	Dopamine
ELISA	Enzyme-linked immunosorbent assay
ERK1/2	Extracellular signal-regulated kinases 1 and 2
GABBR2	Gamma-aminobutyric acid type B receptor subunit 2
Gi/o	G protein inhibitory/olfactory subunits
GNG3	G protein subunit gamma 3
GSH	Glutathione
GSH-Px	Glutathione peroxidase
GSK-3β	Glycogen synthase kinase 3 beta
GSSG	Oxidized glutathione
HE	Hematoxylin–eosin
IACUC	Institutional Animal Care and Use Committee
IL-1β	Interleukin-1β
IL-6	Interleukin-6
iNOS	Inducible nitric oxide synthase
IP3R	Inositol 1,4,5-trisphosphate receptors
ITPR2	Inositol 1,4,5-trisphosphate receptor type 2
KEGG	Kyoto Encyclopedia of Genes and Genomes
MAPKAPK2	Mitogen-activated protein kinase-activated protein kinase 2
MAPT	Microtubule-associated protein tau
MCP-1	Monocyte chemoattractant protein-1
MDA	Malondialdehyde
MSK1	Mitogen- and stress-activated protein kinase 1
NDUFV3	NADH–ubiquinone oxidoreductase core subunit V3
NIOSH	National Institute for Occupational Safety and Health
NMDAR	N-methyl-D-aspartate receptor
OMPM	Oil-mist particulate matter
PDE7B	Phosphodiesterase 7B
PGD_2_	Prostaglandin D_2_
PKA	Protein kinase A
PKC	Protein kinase C
PSMA3/5	Proteasome subunit alpha 3/5
PRAK	p38-regulated/activated protein kinase
ROS	Reactive oxygen species
SNc	Substantia Nigra pars compacta
SOD	Superoxide dismutase
STX1A	Syntaxin 1A
T-AOC	Total antioxidant capacity
TEM	Transmission electron microscopy
TH	Tyrosine hydroxylase
TNF-α	Tumor necrosis factor-α
UQCRC1	Ubiquinol–cytochrome c reductase core protein 1
WB	Western blotting
