# Supplementary material for: Protein Modifications and Metabolic Alterations in the Rat Striatum Following Oil Mist Particulate Matter Exposure Revealed via Untargeted Metabolomics and Phosphoproteomics
Source: Toxics. 2026 Mar 12;14(3):249. doi: 10.3390/toxics14030249 (PMC13030572; doi:10.3390/toxics14030249)
Supplement: Supplementary file 1 [file toxics-14-00249-s001.zip › toxics-4122859-supplementary/toxics-4122859-supplementary.pdf]

Supplementary Material

Protein Modifications and Metabolic Alterations in the Rat Striatum Following Oil Mist Particulate Matter Exposure Revealed *via* Untargeted Metabolomics and Phosphoproteomics

Huipeng Nie[1], Xuan Liu[1], Yue Shi[1], Huanliang Liu[1], Wenqing Lai[1], Kang Li[1], Lei Tian[1], Zhuge Xi[1,*], Bencheng Lin[1,*]

[1] Military Medical Sciences Academy, Academy of Military Sciences, Tianjin Key Laboratory of Risk Assessment and Control Technology for Environment and Food Safety, China.
* Correspondence: zhugexi2003@sina.com (Z.-G.X.); linbencheng123@126.com (B.-C.L.)

Supplementary table

Table S1: List of significantly differential modified peptides in the hierarchical clustering heatmap (from left to right).

Number (from left to right)	Peptide ID and modification information
**1**	ENSRNOP00000017102|TPSPPEEASPLPSPTASPNHTLAPASPAPVRPR|1xTMTpro [N−Term];3xPhospho [T1(50); S3(50); S9(100)]
**2**	ENSRNOP00000001564|DSSSSSSSSSDSDSDGEEHGSDIGPR|1xTMTpro [N−Term];1xPhospho [S4(96.4)]
**3**	ENSRNOP00000048499|AISEELDHALNDMTSI|1xTMTpro [N−Term];1xPhospho [T/S]
**4**	ENSRNOP00000025232|HDSDASPPR|1xTMTpro [N−Term];1xPhospho [S6(100)]
**5**	ENSRNOP00000000804|KGSDSSAPDEER|1xTMTpro [K1];1xTMTpro [N−Term];1xPhospho [S3(100)]
**6**	ENSRNOP00000028380|DGMDTSCSTGSPGAATANR|1xCarbamidomethyl [C7];1xTMTpro [N−Term];1xPhospho [S11(100)]
**7**	ENSRNOP00000002736|RAKSPTPDGSER|1xTMTpro [K3];1xTMTpro [N−Term];2xPhospho [S4(100); S10(98)]
**8**	ENSRNOP00000022813|AGPPPSPGAPSDDDDDDAGATPPAR|1xTMTpro [N−Term];2xPhospho [S11(99.5); T21(100)]
**9**	ENSRNOP00000066704|EGSQGELTPANSQSR|1xTMTpro [N−Term];1xPhospho [S3(100)]
**10**	ENSRNOP00000070910|VVSSTSEEEEAFTEK|1xTMTpro [K15];1xTMTpro [N−Term];1xPhospho [S6(100)]
**11**	ENSRNOP00000028379|LHLESPQPSPKSSPQEAGNVDIWR|1xTMTpro [K11];1xTMTpro [N−Term];3xPhospho [S5(100); S9(95.1); S12(95.1)]

**12**	ENSRNOP00000017265|LSMEDSKSPPPK|2xTMTpro [K7; K12];1xTMTpro [N−Term];1xPhospho [S]
**13**	ENSRNOP00000023460|IAELEEERSQGSTSNSDWMK|1xOxidation [M19];1xTMTpro [K20];1xTMTpro [N−Term];2xPhospho [S9(100); S16(95.3)]
**14**	ENSRNOP00000016420|SGSRPQSPSGDAESR|1xTMTpro [N−Term];2xPhospho [S7(96.7); S]
**15**	ENSRNOP00000018340|AAVVTSPPPTTAPHK|1xTMTpro [K15];1xTMTpro [N−Term];1xPhospho [S6(98.1)]
**16**	ENSRNOP00000070456|NSASSSPVASEAPSR|1xTMTpro [N−Term];1xPhospho [S]
**17**	ENSRNOP00000073375|CLSPDDSTVK|1xCarbamidomethyl [C1];1xTMTpro [K10];1xTMTpro [N−Term];1xPhospho [S3(100)]
**18**	ENSRNOP00000041787|TLSDESLCSGR|1xCarbamidomethyl [C8];1xTMTpro [N−Term];1xPhospho [S3(100)]
**19**	ENSRNOP00000041373|EVSTPDCH|1xCarbamidomethyl [C7];1xTMTpro [N−Term];1xPhospho [T4(100)]
**20**	ENSRNOP00000019214|AMGDEESGDSDGSPKTSPK|1xOxidation [M2];2xTMTpro [K15; K19];1xTMTpro [N−Term];2xPhospho [S7(95.4); S13(99.5)]
**21**	ENSRNOP00000073375|FTDQSLSPEDAESLSVLSVVSPDTTK|1xTMTpro [K26];1xTMTpro [N−Term];1xPhospho [S/T]
**22**	ENSRNOP00000029247|YREGSPLKEESLAR|1xTMTpro [K8];1xTMTpro [N−Term];1xPhospho [S5(100)]
**23**	ENSRNOP00000063720|AAQQTKGSYMEVEDNR|1xOxidation [M10];1xTMTpro [K6];1xTMTpro [N−Term];1xPhospho [S/Y/T]
**24**	ENSRNOP00000065540|EEDEEAESPPEKK|2xTMTpro [K12; K13];1xTMTpro [N−Term];1xPhospho [S8(100)]
**25**	ENSRNOP00000055378|SVSTSPSILPAFLK|1xTMTpro [K14];1xTMTpro [N−Term];2xPhospho [S1(99.9); S5(97.1)]
**26**	ENSRNOP00000063767|RSPPPSPTTQR|1xTMTpro [N−Term];2xPhospho [S2(100); S6(93.8)]
**27**	ENSRNOP00000058049|AEVASQK|1xTMTpro [K7];1xTMTpro [N−Term];1xPhospho [S5(100)]
**28**	ENSRNOP00000041940|STVASCMHR|1xCarbamidomethyl [C6];1xTMTpro [N−Term];1xPhospho [T2(97.4)]
**29**	ENSRNOP00000067418|KPSASLPSPSGSR|1xTMTpro [K1];1xTMTpro [N−Term];1xPhospho [S8(94.5)]
**30**	ENSRNOP00000017102|AFSIQGAPSDSGPLR|1xTMTpro [N−Term];1xPhospho [S11(100)]
**31**	ENSRNOP00000019214|AMGDEESGDSDGSPKTSPK|1xOxidation [M2];2xTMTpro [K15; K19];1xTMTpro [N−Term];3xPhospho [S13(95.5); T16(88.2); S17(99.4)]
**32**	ENSRNOP00000015843|FTASPLEEER|1xTMTpro [N−Term];1xPhospho [S4(100)]

**33**	ENSRNOP00000073375|DLWPMVSPEDTQSLSFSEESPSKETSLDISSK|1xOxidation [M5];2xTMTpro [K23; K32];1xTMTpro [N−Term];2xPhospho [S7(100); S20(93.8)]
**34**	ENSRNOP00000055378|SVSTSPSILPAFLK|1xTMTpro [K14];1xTMTpro [N−Term];2xPhospho [S1(99.9); T4(95)]
**35**	ENSRNOP00000018072|EKSPPDQSAVPNTPPSTPVKLEGGLPQEPTSR|2xTMTpro [K2; K20];1xTMTpro [N−Term];3xPhospho [S3(100); T13(97.2); T17(97.2)]
**36**	ENSRNOP00000023460|DVMSDETNNEETESPSQEFVNITK|1xOxidation [M3];1xTMTpro [K24];1xTMTpro [N−Term];1xPhospho [T12(98.4)]
**37**	ENSRNOP00000044665|VGSLTPPSSPK|1xTMTpro [K11];1xTMTpro [N−Term];2xPhospho [T5(95.7); S9(97.8)]
**38**	ENSRNOP00000039162|SLSDPKPLSPTAEESAK|2xTMTpro [K6; K17];1xTMTpro [N−Term];2xPhospho [S3(99.9); S9(100)]
**39**	ENSRNOP00000073375|GFKSPPCEDFSVTGESEK|1xCarbamidomethyl [C7];2xTMTpro [K3; K18];1xTMTpro [N−Term];1xPhospho [S4(100)]
**40**	ENSRNOP00000023460|VQSLEGEKLSPK|2xTMTpro [K8; K12];1xTMTpro [N−Term];1xPhospho [S10(100)]
**41**	ENSRNOP00000040277|KESLSESR|1xTMTpro [K1];1xTMTpro [N−Term];1xPhospho [S3(100)]
**42**	ENSRNOP00000014359|FYYSSGSSSPTHAK|1xTMTpro [K14];1xTMTpro [N−Term];2xPhospho [S4(50); S5(50)]
**43**	ENSRNOP00000056847|STDSSSYPSPCASPSPPSSGK|1xCarbamidomethyl [C11];1xTMTpro [K21];1xTMTpro [N−Term];2xPhospho [S13(100); S15(100)]
**44**	ENSRNOP00000018694|QSLGESPR|1xTMTpro [N−Term];1xPhospho [S6(100)]
**45**	ENSRNOP00000023460|SRTPVQDHR|1xTMTpro [N−Term];1xPhospho [T3(100)]
**46**	ENSRNOP00000058049|ASQPGPTAESQSSPHR|1xTMTpro [N−Term];1xPhospho [S10(94.3)]
**47**	ENSRNOP00000016194|SETGSTISMSSLER|1xTMTpro [N−Term];1xPhospho [S/T]
**48**	ENSRNOP00000065047|SHYLQRRLPLSFLASSK|1xTMTpro [K17];1xTMTpro [N−Term];3xPhospho [S1(99.5); S11(99.5); S15(93.7)]
**49**	ENSRNOP00000073387|LGSSPTSSCNPTPTK|1xCarbamidomethyl [C9];1xTMTpro [K15];1xTMTpro [N−Term];1xPhospho [S7(97.2)]
**50**	ENSRNOP00000072043|VLSSTINNQVAEADEAAEEEAPDSPGVEK|1xTMTpro [K29];1xTMTpro [N−Term];1xPhospho [S24(100)]
**51**	ENSRNOP00000072175|SSTSPCGTSK|1xCarbamidomethyl [C6];1xTMTpro [K10];1xTMTpro [N−Term];1xPhospho [S4(98)]
**52**	ENSRNOP00000055932|TPSTDEQQQVQEEVK|1xTMTpro [K15];1xTMTpro [N−Term];1xPhospho [S/T]
**53**	ENSRNOP00000056036|LSSPAQSPSQK|1xTMTpro [K11];1xTMTpro [N−Term];2xPhospho [S3(96.8); S9(96.9)]
**54**	ENSRNOP00000075731|ETIKVETPTDIHSEK|2xTMTpro [K4; K15];1xTMTpro [N−Term];1xPhospho [T7(98.8)]

**55**	ENSRNOP00000003867|GQTNNAASASASNST|1xTMTpro [N−Term];1xPhospho [S/T]
**56**	ENSRNOP00000018556|RGSGQDPAGTAPETLEQTEGATP|1xTMTpro [N−Term];1xPhospho [S3(100)]
**57**	ENSRNOP00000026928|ITSPLMEPSSIEK|1xOxidation [M6];1xTMTpro [K13];1xTMTpro [N−Term];1xPhospho [S3(97.5)]
**58**	ENSRNOP00000014359|FYYSSGSSSPTHAK|1xTMTpro [K14];1xTMTpro [N−Term];1xPhospho [S7(96.7)]
**59**	ENSRNOP00000023460|DVMSDETNNEETESPSQEFVNITK|1xOxidation [M3];1xTMTpro [K24];1xTMTpro [N−Term];2xPhospho [S4(100); T7(94.6)]
**60**	ENSRNOP00000023460|TLEVVSPSQSVTGSAGHTPYYQSPTDEK|1xTMTpro [K28];1xTMTpro [N−Term];2xPhospho [S6(100); S23(99.4)]
**61**	ENSRNOP00000038884|QTSSSVDIIPGSCISSNNSPQPLTSR|1xCarbamidomethyl [C13];1xTMTpro [N−Term];1xPhospho [S15(97.1)]
**62**	ENSRNOP00000044583|SSSISSIDKDSK|2xTMTpro [K9; K12];1xTMTpro [N−Term];2xPhospho [S2(94.9); S]
**63**	ENSRNOP00000014359|FYYSSGSSSPTHAK|1xTMTpro [K14];1xTMTpro [N−Term];1xPhospho [S9(98)]
**64**	ENSRNOP00000049690|ELATHAAPLGSYAR|1xTMTpro [N−Term];1xPhospho [S11(100)]
**65**	ENSRNOP00000049838|TVSDNSLSSSK|1xTMTpro [K11];1xTMTpro [N−Term];2xPhospho [S3(97.2); S6(99.9)]
**66**	ENSRNOP00000005519|GSASSGSTTSPTCSPK|1xCarbamidomethyl [C13];1xTMTpro [K16];1xTMTpro [N−Term];1xPhospho [S5(97.2)]
**67**	ENSRNOP00000014359|FYYSSGSSSPTHAK|1xTMTpro [K14];1xTMTpro [N−Term];2xPhospho [S4(97.3); S9(99.9)]
**68**	ENSRNOP00000017599|EERPGAAGGGASAPGSPDETAR|1xTMTpro [N−Term];2xPhospho [S12(100); S16(100)]
**69**	ENSRNOP00000071109|EASPSR|1xTMTpro [N−Term];1xPhospho [S5(98.2)]
**70**	ENSRNOP00000023705|HGASAAPSPPPR|1xTMTpro [N−Term];1xPhospho [S]
**71**	ENSRNOP00000026392|MSESLDTADPAVTGAK|1xTMTpro [K16];1xTMTpro [N−Term];1xPhospho [T13(100)]
**72**	ENSRNOP00000070493|GASSPDMEPSYGGGLFDMVK|1xOxidation [M7];1xTMTpro [K20];1xTMTpro [N−Term];1xPhospho [S4(97.7)]
**73**	ENSRNOP00000062549|DGSPPPAFKPEPPK|2xTMTpro [K9; K14];1xTMTpro [N−Term];1xPhospho [S3(100)]
**74**	ENSRNOP00000014250|QASISGPAPPK|1xTMTpro [K11];1xTMTpro [N−Term];1xPhospho [S5(98.3)]
**75**	ENSRNOP00000055097|YLATASTMDHAR|1xOxidation [M8];1xTMTpro [N−Term];1xPhospho [S6(100)]
**76**	ENSRNOP00000069544|QAAQATGSPHTSPTHGGGR|1xTMTpro [N−Term];2xPhospho [T14(100); T/S]
**77**	ENSRNOP00000074766|ESSPVPSPTSDR|1xTMTpro [N−Term];1xPhospho [S/T]
**78**	ENSRNOP00000020123|SNSQENVEASHPSQDAK|1xTMTpro [K17];1xTMTpro

	[N−Term];1xPhospho [S3(97.9)]
**79**	ENSRNOP00000070493|ATTSASASPTLR|1xTMTpro [N−Term];1xPhospho [S/T]
**80**	ENSRNOP00000042392|SPVVSGDTSPR|1xTMTpro [N−Term];2xPhospho [T8(99.8); S9(99.8)]
**81**	ENSRNOP00000006443|RRGSGSVDETLFALPAASEPVIPSSAEK|1xTMTpro [K28];1xTMTpro [N−Term];2xPhospho [S4(97.4); S6(97.4)]
**82**	ENSRNOP00000070493|RATTSASASPTLR|1xTMTpro [N−Term];1xPhospho [S9(99.9)]
**83**	ENSRNOP00000057375|NSSMGSPVNQQPK|1xTMTpro [K13];1xTMTpro [N−Term];1xPhospho [S6(97.5)]
**84**	ENSRNOP00000006162|SSSVSSVNSPQQNSAK|1xTMTpro [K16];1xTMTpro [N−Term];2xPhospho [S6(96.3); S]
**85**	ENSRNOP00000043328|LAEMYGGDDSDRD|1xOxidation [M4];1xTMTpro [N−Term];1xPhospho [S10(100)]
**86**	ENSRNOP00000027337|FSIGSDEDDSPGLSIK|1xTMTpro [K16];1xTMTpro [N−Term];1xPhospho [S5(100)]
**87**	ENSRNOP00000006443|RGSGSVDETLFALPAASEPVIPSSAEK|1xTMTpro [K27];1xTMTpro [N−Term];1xPhospho [S3(97.4)]
**88**	ENSRNOP00000068599|RAPATARVSPPESPR|1xTMTpro [N−Term];2xPhospho [S9(96.9); S13(100)]
**89**	ENSRNOP00000027682|QAEAVTSPR|1xTMTpro [N−Term];1xPhospho [T/S]
**90**	ENSRNOP00000073548|SSSVGSSSSYPISSAVPR|1xTMTpro [N−Term];1xPhospho [S2(97.4)]
**91**	ENSRNOP00000023478|SSSNESFSSNQSADSAPDEETLALR|1xTMTpro [N−Term];1xPhospho [S/T]
**92**	ENSRNOP00000019214|AMGDEESGDSDGSPKTSPK|1xOxidation [M2];2xTMTpro [K15; K19];1xTMTpro [N−Term];2xPhospho [T16(99.7); S17(100)]
**93**	ENSRNOP00000018849|SVGSPEESTER|1xTMTpro [N−Term];1xPhospho [S4(100)]
**94**	ENSRNOP00000068198|VARPQILEPRPQSPDLCDDDVEFR|1xCarbamidomethyl [C17];1xTMTpro [N−Term];1xPhospho [S13(100)]
**95**	ENSRNOP00000022625|SNSPENTR|1xTMTpro [N−Term];1xPhospho [S3(100)]
**96**	ENSRNOP00000023460|TTRTPEEGGYSYEISEK|1xTMTpro [K17];1xTMTpro [N−Term];1xPhospho [T4(98.2)]
**97**	ENSRNOP00000027682|QAEAVTSPR|1xTMTpro [N−Term];1xPhospho [S7(100)]
**98**	ENSRNOP00000016555|STSPPPSPEVWAESR|1xTMTpro [N−Term];1xPhospho [S3(98.5)]
**99**	ENSRNOP00000053288|GSNSDSPPGHSAPVPSPER|1xTMTpro [N−Term];1xPhospho [S6(99.9)]
**100**	ENSRNOP00000064597|APSVSPPVPPTK|1xTMTpro [K12];1xTMTpro [N−Term];1xPhospho [S/T]
**101**	ENSRNOP00000074621|DSGHEESDQTDSEHDVQR|1xTMTpro [N−Term];3xPhospho [S7(100); T10(100); S12(100)]

**102**	ENSRNOP00000016070|LADVSAPSGGPPPPHSPYSGPPSRGSPVR|1xTMTpro [N−Term];2xPhospho [S23(93.1); S26(93.1)]
**103**	ENSRNOP00000021189|ESSRVSFEDQAPTME|1xTMTpro [N−Term];1xPhospho [S/T]
**104**	ENSRNOP00000003320|DLEGTIR|1xTMTpro [N−Term];1xPhospho [T5(100)]
**105**	ENSRNOP00000011604|TLDVKSPEAK|2xTMTpro [K5; K10];1xTMTpro [N−Term];1xPhospho [S6(100)]
**106**	ENSRNOP00000067534|GPSPLVTMTPAVPAVTPVDEESSDGEPDQEAVQR|1xTMTpro [N−Term];2xPhospho [T7(94.2); T/S]
**107**	ENSRNOP00000023460|TTRTPEEGGYSYEISEK|1xTMTpro [K17];1xTMTpro [N−Term];1xPhospho [T1(97.7)]
**108**	ENSRNOP00000068918|SETSPVAPR|1xTMTpro [N−Term];1xPhospho [T/S]
**109**	ENSRNOP00000035714|RVTNTQESQNASPK|1xTMTpro [K14];1xTMTpro [N−Term];1xPhospho [S12(100)]
**110**	ENSRNOP00000019601|AESGDSLSSEDRDLLYSIDAYR|1xTMTpro [N−Term];2xPhospho [S3(100); S6(99.8)]
**111**	ENSRNOP00000073375|FTDQSLSPEDAESLSVLSVVSPDTTKQEATPR|1xTMTpro [K26];1xTMTpro [N−Term];3xPhospho [S5(100); S7(100); S21(94.2)]
**112**	ENSRNOP00000067467|ELVSSSSSGSDSDSEVEK|1xTMTpro [K18];1xTMTpro [N−Term];3xPhospho [S7(92.2); S12(94.8); S14(99.8)]
**113**	ENSRNOP00000053288|GSNSDSPPGHSAPVPSPERR|1xTMTpro [N−Term];2xPhospho [S4(93.7); S16(100)]
**114**	ENSRNOP00000027682|QAEAVTSPR|1xTMTpro [N−Term];1xPhospho [T6(98.1)]
**115**	ENSRNOP00000023645|GSDYSWSYQTPPSSPSSSSSR|1xTMTpro [N−Term];1xPhospho [S14(89.2)]
**116**	ENSRNOP00000060972|PEPSRSTPAPK|1xTMTpro [K11];1xTMTpro [N−Term];1xPhospho [S/T]
**117**	ENSRNOP00000055097|TTHYGSLPQKSQR|1xTMTpro [K10];1xTMTpro [N−Term];2xPhospho [S6(99.9); Y/S/T]
**118**	ENSRNOP00000006443|EDKIKESETFSDSSPIEIIDEFPTFVSAK|3xTMTpro [K3; K5; K29];1xTMTpro [N−Term];2xPhospho [S/T]
**119**	ENSRNOP00000041403|TISNPEGSPSRSPR|1xTMTpro [N−Term];1xPhospho [S10(97.7)]
**120**	ENSRNOP00000016555|STSPPPSPEVWAESR|1xTMTpro [N−Term];1xPhospho [T2(94.7)]
**121**	ENSRNOP00000058819|VSSVSSDEIWL|1xTMTpro [N−Term];2xPhospho [S3(97.7); S5(99.9)]
**122**	ENSRNOP00000071379|SSPATTVTSPNSTPAK|1xTMTpro [K16];1xTMTpro [N−Term];1xPhospho [T8(94.6)]
**123**	ENSRNOP00000006443|EDKIKESETFSDSSPIEIIDEFPTFVSAK|3xTMTpro [K3; K5; K29];1xTMTpro [N−Term];2xPhospho [S13(49.3); S14(49.3)]
**124**	ENSRNOP00000042949|SRTLSQSSESGTLPSGPPGHTMEVSC|1xCarbamidomethyl [C26];1xTMTpro [N−Term];2xPhospho [T3(97.2); S5(100)]

**125**	ENSRNOP00000025740|RESDESGESAPEEGGEGAR|1xTMTpro [N−Term];2xPhospho [S6(100); S9(100)]
**126**	ENSRNOP00000060346|EMEAPKSPGTAR|1xTMTpro [K6];1xTMTpro [N−Term];1xPhospho [S7(100)]
**127**	ENSRNOP00000071171|SHSYTSDN|1xTMTpro [N−Term];1xPhospho [T/S/Y]
**128**	ENSRNOP00000037320|TGDQESEDEL|1xTMTpro [N−Term];1xPhospho [S6(100)]
**129**	ENSRNOP00000047081|SQSPHYFR|1xTMTpro [N−Term];1xPhospho [S3(100)]
**130**	ENSRNOP00000016555|ECSLSPKSTSPPPSPEVWAESR|1xCarbamidomethyl [C2];1xTMTpro [K7];1xTMTpro [N−Term];2xPhospho [T9(66.7); S10(66.7)]
**131**	ENSRNOP00000072364|LQEEFEAALGGSDPDSLANDTDKDTDLSVR|1xTMTpro [K23];1xTMTpro [N−Term];2xPhospho [S12(100); S16(100)]
**132**	ENSRNOP00000074488|VGGAKPAGGDFGEVLNSTANATSTTTTEPPPEQTTESP|1xTMTpro [K5];1xTMTpro [N−Term];1xPhospho [S37(100)]
**133**	ENSRNOP00000046923|RSSPVPR|1xTMTpro [N−Term];1xPhospho [S]
**134**	ENSRNOP00000064525|SAAQVSSSR|1xTMTpro [N−Term];1xPhospho [S8(97.7)]
**135**	ENSRNOP00000042876|HGSSPSTGASSR|1xTMTpro [N−Term];1xPhospho [S/T]
**136**	ENSRNOP00000019214|AMGDEESGDSDGSPKTSPK|2xTMTpro [K15; K19];1xTMTpro [N−Term];4xPhospho [S7(100); S10(100); S13(100); S17(95.2)]
**137**	ENSRNOP00000021289|SKSEMNYIDGEK|2xTMTpro [K2; K12];1xTMTpro [N−Term];1xPhospho [S3(100)]
**138**	ENSRNOP00000010753|ESLKEEDESDDDNM|1xTMTpro [K4];1xTMTpro [N−Term];1xPhospho [S9(100)]
**139**	ENSRNOP00000050996|TCPSPKEACQEPASRPEVNR|2xCarbamidomethyl [C2; C9];1xTMTpro [K6];1xTMTpro [N−Term];1xPhospho [S4(100)]
**140**	ENSRNOP00000074184|RYSPVAK|1xTMTpro [K7];1xTMTpro [N−Term];1xPhospho [S3(100)]
**141**	ENSRNOP00000011604|SPAVAKSPAEVK|2xTMTpro [K6; K12];1xTMTpro [N−Term];1xPhospho [S7(100)]
**142**	ENSRNOP00000011604|SPGEAKSPAEAKSPAEVK|3xTMTpro [K6; K12; K18];1xTMTpro [N−Term];2xPhospho [S7(100); S13(100)]
**143**	ENSRNOP00000072307|SESDSSPDSKPK|2xTMTpro [K10; K12];1xTMTpro [N−Term];2xPhospho [S5(97.9); S9(100)]
**144**	ENSRNOP00000035155|SASPERMD|1xTMTpro [N−Term];1xPhospho [S3(100)]
**145**	ENSRNOP00000037989|SGSVNEASCPTEQTR|1xCarbamidomethyl [C9];1xTMTpro [N−Term];1xPhospho [S/T]
**146**	ENSRNOP00000020219|EHLGQGSSQEMEK|1xTMTpro [K13];1xTMTpro [N−Term];1xPhospho [S8(100)]
**147**	ENSRNOP00000049754|QKEDVEGVGTSDGEGAAGLSSDPK|2xTMTpro [K2; K24];1xTMTpro [N−Term];1xPhospho [T/S]
**148**	ENSRNOP00000046690|GSDSDSEGDNPEK|1xTMTpro [K13];1xTMTpro [N−Term];1xPhospho [S]
**149**	ENSRNOP00000013301|GHSQEK|1xTMTpro [K6];1xTMTpro

	[N−Term];1xPhospho [S3(100)]
**150**	ENSRNOP00000075014|QIASDSPHASPK|1xTMTpro [K12];1xTMTpro [N−Term];2xPhospho [S10(100); S]
**151**	ENSRNOP00000075849|KDDQSPLDIK|2xTMTpro [K1; K10];1xTMTpro [N−Term];1xPhospho [S5(100)]
**152**	ENSRNOP00000011604|SPVEAKSPAEVK|2xTMTpro [K6; K12];1xTMTpro [N−Term];1xPhospho [S7(100)]
**153**	ENSRNOP00000069847|VSDSESEDPQKGPASDSEAEDASR|1xTMTpro [K11];1xTMTpro [N−Term];2xPhospho [S4(95.5); S17(97.7)]
**154**	ENSRNOP00000022351|SHSLEGPSK|1xTMTpro [K9];1xTMTpro [N−Term];1xPhospho [S3(99.2)]
**155**	ENSRNOP00000052955|GASASPQGRQSPSPSTRPIR|1xTMTpro [N−Term];3xPhospho [S5(96.4); S11(100); S13(93)]
**156**	ENSRNOP00000052955|RYSPSPPPK|1xTMTpro [K9];1xTMTpro [N−Term];2xPhospho [S3(100); S5(100)]
**157**	ENSRNOP00000062321|GSTESCNTTTEDEDLKVR|1xCarbamidomethyl [C6];1xTMTpro [K16];1xTMTpro [N−Term];2xPhospho [T9(97.6); T10(99.9)]
**158**	ENSRNOP00000005623|VGVEELSEEDQNEHR|1xTMTpro [N−Term];1xPhospho [S7(100)]
**159**	ENSRNOP00000052268|LCDKPASPR|1xCarbamidomethyl [C2];1xTMTpro [K4];1xTMTpro [N−Term];1xPhospho [S7(100)]
**160**	ENSRNOP00000072307|GPSPEGSSSSESSPEHPPK|1xTMTpro [K19];1xTMTpro [N−Term];2xPhospho [S12(100); S13(99.9)]
**161**	ENSRNOP00000052955|VSSSRSVSGSPEPTAK|1xTMTpro [K16];1xTMTpro [N−Term];3xPhospho [S2(90.2); S8(100); S10(100)]
**162**	ENSRNOP00000043615|RDSVLAASR|1xTMTpro [N−Term];1xPhospho [S3(100)]
**163**	ENSRNOP00000052955|GASASPQGRQSPSPSTRPIR|1xTMTpro [N−Term];3xPhospho [S3(95.2); S5(95.2); S/T]
**164**	ENSRNOP00000042235|DKGSPHSEGSDR|1xTMTpro [K2];1xTMTpro [N−Term];1xPhospho [S4(100)]
**165**	ENSRNOP00000041940|ESSESTNTTIEDEDTKVR|1xTMTpro [K16];1xTMTpro [N−Term];2xPhospho [S3(99.9); T8(100)]
**166**	ENSRNOP00000073084|SKEFVSSDESSSGENK|2xTMTpro [K2; K16];1xTMTpro [N−Term];1xPhospho [S]
**167**	ENSRNOP00000027985|ASLPTCLLMCVCLAQAFGYRLR|3xCarbamidomethyl [C6; C10; C12];1xTMTpro [N−Term];2xPhospho [S/T/Y]
**168**	ENSRNOP00000011604|SPVEAKSPAEAK|2xTMTpro [K6; K12];1xTMTpro [N−Term];1xPhospho [S7(100)]
**169**	ENSRNOP00000070910|FEDYLREPAPGDPGCGPGELRPPSPTSPEGPDTGQK|1xCarbamidomethyl [C15];1xTMTpro [K36];1xTMTpro [N−Term];2xPhospho [S24(95.6); S27(95.6)]
**170**	ENSRNOP00000052955|EARSPQPNK|1xTMTpro [K9];1xTMTpro [N−Term];1xPhospho [S4(100)]

**171**	ENSRNOP00000052955|AASPSPQSVR|1xTMTpro [N−Term];1xPhospho [S]
**172**	ENSRNOP00000011604|SPASVKSPGEAK|2xTMTpro [K6; K12];1xTMTpro [N−Term];2xPhospho [S4(99.9); S7(99.9)]
**173**	ENSRNOP00000052955|AASPSPQSVR|1xTMTpro [N−Term];1xPhospho [S5(100)]
**174**	ENSRNOP00000052955|KAASPSPQSVR|1xTMTpro [K1];1xTMTpro [N−Term];1xPhospho [S4(97.5)]
**175**	ENSRNOP00000054654|SHSYLR|1xTMTpro [N−Term];1xPhospho [S3(100)]
**176**	ENSRNOP00000022351|ATQPSLTELTTLK|1xTMTpro [K13];1xTMTpro [N−Term];1xPhospho [T2(98.7)]
**177**	ENSRNOP00000013301|KGDTDHSRSPSR|1xTMTpro [K1];1xTMTpro [N−Term];2xPhospho [S9(97.4); S/T]
**178**	ENSRNOP00000050121|GRVSPSDADSTVSEESSERDVGEK|1xTMTpro [K24];1xTMTpro [N−Term];1xPhospho [S4(100)]
**179**	ENSRNOP00000052955|APQTSSPPPVRR|1xTMTpro [N−Term];1xPhospho [S/T]
**180**	ENSRNOP00000022351|AVSEVSINR|1xTMTpro [N−Term];2xPhospho [S3(100); S6(100)]
**181**	ENSRNOP00000008560|KQGGSPDEPDCK|1xCarbamidomethyl [C11];2xTMTpro [K1; K12];1xTMTpro [N−Term];1xPhospho [S5(100)]
**182**	ENSRNOP00000002429|RRSPSPYYSR.|1xTMTpro [N−Term];2xPhospho [S3(100); S/Y]
**183**	ENSRNOP00000025878|HSSLPTESDEDIAPAQR|1xTMTpro [N−Term];1xPhospho [S2(98.1)]
**184**	ENSRNOP00000034166|AMGDEDSDESGGGSPKPSPK|2xTMTpro [K16; K20];1xTMTpro [N−Term];1xPhospho [S]
**185**	ENSRNOP00000006637|GGQDDDDAETGLTEGEGEGEEEKEPENLGK|2xTMTpro [K23; K30];1xTMTpro [N−Term];1xPhospho [T13(100)]
**186**	ENSRNOP00000025788|KAAAQTQPADSSADSSEESDSSSEEEKKTPAK|4xTMTpro [K1; K27; K28; K32];1xTMTpro [N−Term];2xPhospho [S22(94.2); S23(94.2)]
**187**	ENSRNOP00000069267|GGGSGGGDESEGEEVDED|1xTMTpro [N−Term];1xPhospho [S4(100)]
**188**	ENSRNOP00000073898|VLHAQCHSTPDSAEDVRK|1xCarbamidomethyl [C6];1xTMTpro [K18];1xTMTpro [N−Term];1xPhospho [T9(96.9)]
**189**	ENSRNOP00000001564|DSSSSSSSSSDSDSDGEEHGSDIGPR|1xTMTpro [N−Term];1xPhospho [S6(92.7)]
**190**	ENSRNOP00000041993|SSDWSSEEEEPVRK|1xTMTpro [K14];1xTMTpro [N−Term];2xPhospho [S5(100); S6(100)]
**191**	ENSRNOP00000001564|DSSSSSSSSSDSDSDGEEHGSDIGPR|1xTMTpro [N−Term];2xPhospho [S4(93.4); S15(99.9)]
**192**	ENSRNOP00000022892|TGTAEMSSILEER|1xTMTpro [N−Term];1xPhospho [S/T]
**193**	ENSRNOP00000052955|RASPSPPPKR|1xTMTpro [K9];1xTMTpro [N−Term];2xPhospho [S3(100); S5(100)]

**194**	ENSRNOP00000052955|RYSPSPPPKR|1xTMTpro [K9];1xTMTpro [N−Term];2xPhospho [S3(100); S5(100)]
**195**	ENSRNOP00000066239|IGAGPQTVETGSETEDEAIFESLIWAAK|1xTMTpro [K28];1xTMTpro [N−Term];3xPhospho [T10(97.6); S12(97.6); S22(97.6)]
**196**	ENSRNOP00000062321|QSSAPASPAASAAGLAGQAAK|1xTMTpro [K21];1xTMTpro [N−Term];1xPhospho [S3(97.5)]
**197**	ENSRNOP00000001564|DSSSSSSSSSDSDSDGEEHGSDIGPR|1xTMTpro [N−Term];3xPhospho [S13(98.5); S15(100); S]
**198**	ENSRNOP00000061603|ESPVPPSSTPPPPPEISPSTSLK|1xTMTpro [K23];1xTMTpro [N−Term];2xPhospho [T9(90.2); S/T]
**199**	ENSRNOP00000063739|GGPAEEPSPRGSPR|1xTMTpro [N−Term];1xPhospho [S]
**200**	ENSRNOP00000052955|AASPSPQSVRR|1xTMTpro [N−Term];1xPhospho [S5(99.8)]
**201**	ENSRNOP00000022625|QQGVLQSSPKNAEGSTVTCTGSIR|1xCarbamidomethyl [C19];1xTMTpro [K10];1xTMTpro [N−Term];1xPhospho [S/T]
**202**	ENSRNOP00000011604|SPAEAKSPAEVKSPVEAK|3xTMTpro [K6; K12; K18];1xTMTpro [N−Term];1xPhospho [S7(100)]
**203**	ENSRNOP00000001957|ARPASTSPSPGAHGR|1xTMTpro [N−Term];2xPhospho [S5(99.9); S7(97.5)]
**204**	ENSRNOP00000010216|CDHESSPGTDEDKSG|1xCarbamidomethyl [C1];1xTMTpro [K13];1xTMTpro [N−Term];2xPhospho [S14(100); S/T]
**205**	ENSRNOP00000075731|SKSESDASSLDAK|2xTMTpro [K2; K13];1xTMTpro [N−Term];1xPhospho [S]
**206**	ENSRNOP00000064723|SNSWQGNVGGNK|1xTMTpro [K12];1xTMTpro [N−Term];1xPhospho [S3(98.6)]
**207**	ENSRNOP00000018711|AQSGPVR|1xTMTpro [N−Term];1xPhospho [S3(100)]
**208**	ENSRNOP00000045958|YVLTSPR|1xTMTpro [N−Term];1xPhospho [T4(97.8)]
**209**	ENSRNOP00000070347|GYSSPEPDVQDSSGSEAQSVKPSTR|1xTMTpro [K21];1xTMTpro [N−Term];3xPhospho [S3(97.5); S4(34.7); S19(96.2)]
**210**	ENSRNOP00000072307|SGSESSVEQKTLTR|1xTMTpro [K10];1xTMTpro [N−Term];2xPhospho [S5(100); S6(100)]
**211**	ENSRNOP00000040699|TAKDSDDDDDVTVTVDRDRFMDEFFEQVEEIR|1xTMTpro [K3];1xTMTpro [N−Term];1xPhospho [S5(100)]
**212**	ENSRNOP00000052955|AASPSPQSVRR|1xTMTpro [N−Term];2xPhospho [S3(100); S5(100)]
**213**	ENSRNOP00000075849|KSEVQAHSPSR|1xTMTpro [K1];1xTMTpro [N−Term];1xPhospho [S8(98.5)]
**214**	ENSRNOP00000013570|SPEIHR|1xTMTpro [N−Term];1xPhospho [S1(100)]
**215**	ENSRNOP00000002429|SGSAHGSGK|1xTMTpro [K9];1xTMTpro [N−Term];1xPhospho [S3(100)]
**216**	ENSRNOP00000027986|GIPLPTGDTSPEPELLPGDPLPPPKEVINGNIK|2xTMTpro [K25; K33];1xTMTpro [N−Term];1xPhospho [S10(93.1)]
**217**	ENSRNOP00000011604|SPVEAKSPAEVK|2xTMTpro [K6; K12];1xTMTpro

	[N−Term];1xPhospho [S1(100)]
**218**	ENSRNOP00000054947|CIELSCGSVRK|2xCarbamidomethyl [C1; C6];1xTMTpro [K11];1xTMTpro [N−Term];1xPhospho [S8(100)]
**219**	ENSRNOP00000015122|PFSNSHNTQK|1xTMTpro [K10];1xTMTpro [N−Term];1xPhospho [S3(100)]
**220**	ENSRNOP00000011604|SPAEAKSPVEVKSPASVKSPSEAK|4xTMTpro [K6; K12; K18; K24];1xTMTpro [N−Term];3xPhospho [S7(100); S16(99.8); S19(95.2)]
**221**	ENSRNOP00000045830|STGQLNVSPGTPSGSTATAER|1xTMTpro [N−Term];1xPhospho [S/T]
**222**	ENSRNOP00000041940|ESSESTNTTIEDEDTKVRK|2xTMTpro [K16; K19];1xTMTpro [N−Term];3xPhospho [T6(96.5); T8(100); T9(100)]
**223**	ENSRNOP00000075849|KSEVQAHSPSR|1xTMTpro [K1];1xTMTpro [N−Term];1xPhospho [S10(98.4)]
**224**	ENSRNOP00000069092|SLGGQQGSPK|1xTMTpro [K10];1xTMTpro [N−Term];1xPhospho [S8(100)]
**225**	ENSRNOP00000022351|SLDSLDPAGLLTSPKFR|1xTMTpro [K15];1xTMTpro [N−Term];1xPhospho [S13(98)]
**226**	ENSRNOP00000052955|APQTSSPPPVRR|1xTMTpro [N−Term];1xPhospho [S6(97.8)]
**227**	ENSRNOP00000024867|ADPVLLNNHSNLKPAPTVPAAPSSPDTTSEPK|2xTMTpro [K13; K32];1xTMTpro [N−Term];1xPhospho [S23(95.7)]
**228**	ENSRNOP00000022603|VFDKDGNGYISAAELR|1xTMTpro [K4];1xTMTpro [N−Term];1xPhospho [S11(100)]
**229**	ENSRNOP00000012415|GVNFAEEPMRSDSENGEEEEAAEAGAFNAPVINR|1xTMTpro [N−Term];2xPhospho [S11(100); S13(100)]
**230**	ENSRNOP00000071203|MGPSSSPIPSPSPSPTDSKR|1xTMTpro [K19];1xTMTpro [N−Term];1xPhospho [S14(100)]
**231**	ENSRNOP00000034166|AMGDEDSDESGGGSPKPSPK|2xTMTpro [K16; K20];1xTMTpro [N−Term];2xPhospho [S7(98); S]
**232**	ENSRNOP00000015893|KEESEEESEDDMGFGLFD|1xTMTpro [K1];1xTMTpro [N−Term];1xPhospho [S4(100)]
**233**	ENSRNOP00000058819|ENMSLPSNLQLNDLTPDIR|1xTMTpro [N−Term];1xPhospho [S4(100)]
**234**	ENSRNOP00000071049|ESSPPREEAPPPPPPTEDSCAK|1xCarbamidomethyl [C20];1xTMTpro [K22];1xTMTpro [N−Term];1xPhospho [S2(97)]
**235**	ENSRNOP00000011573|ITELDKDLEEVTMQLQDTPEKTTYIK|3xTMTpro [K6; K21; K26];1xTMTpro [N−Term];1xPhospho [T18(91.4)]
**236**	ENSRNOP00000011604|SPGEAKSPAEAK|2xTMTpro [K6; K12];1xTMTpro [N−Term];1xPhospho [S7(100)]
**237**	ENSRNOP00000019077|ERSPPLTPK|1xTMTpro [K9];1xTMTpro [N−Term];1xPhospho [S3(100)]
**238**	ENSRNOP00000004699|GQPVLTPPDQLVIANIDQSDFEGFSYVNPQFVHPILQSAV|1xTMTpro [N−Term];1xPhospho [T6(100)]
**239**	ENSRNOP00000002429|SGSAHGSGK|1xTMTpro [K9];1xTMTpro

	[N−Term];1xPhospho [S]
**240**	ENSRNOP00000028119|ETMSGQTAFLGSPESR|1xTMTpro [N−Term];1xPhospho [S12(100)]
**241**	ENSRNOP00000034166|AMGDEDSDESGGGSPKPSPK|2xTMTpro [K16; K20];1xTMTpro [N−Term];3xPhospho [S7(100); S10(99.9); S18(100)]
**242**	ENSRNOP00000015205|RDSDMTGHIQQPGGR|1xTMTpro [N−Term];1xPhospho [S3(100)]
**243**	ENSRNOP00000038747|ESPESDTGSATTSSDDIKPR|1xTMTpro [K18];1xTMTpro [N−Term];2xPhospho [T12(91.5); S14(97.1)]
**244**	ENSRNOP00000075039|AAETVPDLPSPPTEAPAPASNTSNR|1xTMTpro [N−Term];1xPhospho [T/S]
**245**	ENSRNOP00000044696|RLSRTDLTDYLSR|1xTMTpro [N−Term];1xPhospho [S3(100)]
**246**	ENSRNOP00000024867|SASSPKPDTKVPQATAEAK|3xTMTpro [K6; K10; K19];1xTMTpro [N−Term];1xPhospho [S4(96.9)]
**247**	ENSRNOP00000059742|KQDSDEMPFGCIK|1xCarbamidomethyl [C11];2xTMTpro [K1; K13];1xTMTpro [N−Term];1xPhospho [S4(100)]
**248**	ENSRNOP00000059423|SHSSPSLNPDASPVTAK|1xTMTpro [K17];1xTMTpro [N−Term];2xPhospho [S4(98.9); S/T]
**249**	ENSRNOP00000045830|NSDLFTVLSRSSSPDLSSSSK|1xTMTpro [K21];1xTMTpro [N−Term];2xPhospho [S11(97.8); S13(97.8)]
**250**	ENSRNOP00000068316|RASSESTR|1xTMTpro [N−Term];1xPhospho [S/T]
**251**	ENSRNOP00000015893|KEESEESEDDMGFGLFD|1xTMTpro [K1];1xTMTpro [N−Term];2xPhospho [S4(100); S7(100)]
**252**	ENSRNOP00000073205|TSQSEETR|1xTMTpro [N−Term];1xPhospho [S2(99.9)]
**253**	ENSRNOP00000023460|IAELEEERSQGSTSNSDWMK|1xTMTpro [K20];1xTMTpro [N−Term];1xPhospho [S16(99.9)]
**254**	ENSRNOP00000069913|VGDTSLDPNDFDFTVTGRGSPSRR|1xTMTpro [N−Term];1xPhospho [T14(91.8)]
**255**	ENSRNOP00000013184|ETQTPLATHQSEEDEEDEEMVEPK|1xTMTpro [K24];1xTMTpro [N−Term];1xPhospho [S11(98.7)]
**256**	ENSRNOP00000001564|DSSSSSSSSSSDSDSDGEEHGSDIGPR|1xTMTpro [N−Term];1xPhospho [S15(99.9)]
**257**	ENSRNOP00000027986|GIPLPTGDTSPEPELLPGDPLPPPK|1xTMTpro [K25];1xTMTpro [N−Term];1xPhospho [T/S]
**258**	ENSRNOP00000054654|SDVETATDSDTESR|1xTMTpro [N−Term];2xPhospho [T7(100); S9(100)]
**259**	ENSRNOP00000042392|SGYSSPGSPGTPGSRSR|1xTMTpro [N−Term];1xPhospho [T11(95.3)]
**260**	ENSRNOP00000038037|GYTSDSEVYTDHGRPGK|1xTMTpro [K17];1xTMTpro [N−Term];1xPhospho [T3(98)]
**261**	ENSRNOP00000023705|HGASAAPSPPPR|1xTMTpro [N−Term];1xPhospho [S8(99.9)]
**262**	ENSRNOP00000073548|SSSVGSSSSYPISSAVPR|1xTMTpro

	[N−Term];1xPhospho [S1(95)]
**263**	ENSRNOP00000034166|APSMEGTTGK|1xTMTpro [K10];1xTMTpro [N−Term];1xPhospho [S3(100)]
**264**	ENSRNOP00000021178|SSASFSTTAVSAR|1xTMTpro [N−Term];1xPhospho [S11(100)]
**265**	ENSRNOP00000034166|AMGDEDSDESGGGSPKPSPK|2xTMTpro [K16; K20];1xTMTpro [N−Term];3xPhospho [S7(100); S10(100); S14(100)]
**266**	ENSRNOP00000075849|DEFTAEKEASPPSSADK|2xTMTpro [K7; K17];1xTMTpro [N−Term];1xPhospho [S10(100)]
**267**	ENSRNOP00000025294|EVKSSPSESPLMEK|2xTMTpro [K3; K14];1xTMTpro [N−Term];1xPhospho [S5(98.2)]
**268**	ENSRNOP00000022351|GGVNGGKASPDGSQTVR|1xTMTpro [K7];1xTMTpro [N−Term];1xPhospho [S9(100)]
**269**	ENSRNOP00000052955|HRPSSPATPPPK|1xTMTpro [K12];1xTMTpro [N−Term];2xPhospho [T8(100); S]
**270**	ENSRNOP00000027985|ASLPTCLLMCVCLAQAFGYRLR|3xCarbamidomethyl [C6; C10; C12];1xTMTpro [N−Term];1xPhospho [S/T/Y]
**271**	ENSRNOP00000034166|RDTDSDTQDANDSSCK|1xCarbamidomethyl [C15];1xTMTpro [K16];1xTMTpro [N−Term];2xPhospho [T3(100); S5(100)]
**272**	ENSRNOP00000027985|ASLPTCLLMCVCLAQAFGYRLR|3xCarbamidomethyl [C6; C10; C12];1xTMTpro [N−Term];1xPhospho [T5(94)]
**273**	ENSRNOP00000011604|SPASVKSPSEAK|2xTMTpro [K6; K12];1xTMTpro [N−Term];1xPhospho [S4(100)]
**274**	ENSRNOP00000017892|SPNEVDK|1xTMTpro [K7];1xTMTpro [N−Term];1xPhospho [S1(100)]
**275**	ENSRNOP00000026385|RPPESPPIVEEWNSR|1xTMTpro [N−Term];1xPhospho [S5(100)]
**276**	ENSRNOP00000054654|SDVETATDSDTESR|1xTMTpro [N−Term];2xPhospho [T5(97.9); S9(100)]
**277**	ENSRNOP00000059915|GHTSPCGMK|1xCarbamidomethyl [C6];1xTMTpro [K9];1xTMTpro [N−Term];1xPhospho [T/S]
**278**	ENSRNOP00000071072|GSEDSPPKHSNNNESHSSR|1xTMTpro [K8];1xTMTpro [N−Term];1xPhospho [S5(99.7)]
**279**	ENSRNOP00000067467|PKSKELVSSSSSGSDSDSEVEK|3xTMTpro [K2; K4; K22];1xTMTpro [N−Term];2xPhospho [S3(100); S18(100)]
**280**	ENSRNOP00000022351|SHSYVR|1xTMTpro [N−Term];1xPhospho [S3(100)]
**281**	ENSRNOP00000071222|SSASESR|1xTMTpro [N−Term];1xPhospho [S4(100)]
**282**	ENSRNOP00000044081|TYFSHIDVSPGSAQVK|1xTMTpro [K16];1xTMTpro [N−Term];1xPhospho [S9(99.9)]
**283**	ENSRNOP00000059915|GHTSPCGMK|1xCarbamidomethyl [C6];1xTMTpro [K9];1xTMTpro [N−Term];1xPhospho [T3(98.8)]
**284**	ENSRNOP00000075849|ASQPSPPAHEAGYSTLAQSYTSDHPSELPEEPSSPQER|1xTMTpro [N−Term];1xPhospho [S/Y/T]
**285**	ENSRNOP00000052955|RRSPTPPPR|1xTMTpro [N−Term];2xPhospho [S3(100);

	T5(100)]
**286**	ENSRNOP00000016712|EGPIGGESDSEEVR|1xTMTpro [N−Term];1xPhospho [S8(100)]
**287**	ENSRNOP00000038747|ESPESDTGSATTSSDDIKPR|1xTMTpro [K18];1xTMTpro [N−Term];1xPhospho [T7(96.9)]
**288**	ENSRNOP00000054654|SGSYIK|1xTMTpro [K6];1xTMTpro [N−Term];1xPhospho [S3(100)]
**289**	ENSRNOP00000034166|ANSWQLVETPEKRK|2xTMTpro [K12; K14];1xTMTpro [N−Term];1xPhospho [T9(100)]
**290**	ENSRNOP00000052955|HRPSSPATPPPK|1xTMTpro [K12];1xTMTpro [N−Term];2xPhospho [S5(97.9); T8(100)]
**291**	ENSRNOP00000052955|VSVSPGR|1xTMTpro [N−Term];1xPhospho [S4(100)]
**292**	ENSRNOP00000052955|SSASLSGSSSSSSSSRSR|5xPhospho [S9; S10; S11; S12; S13];1xTMTpro [N−Term]
**293**	ENSRNOP00000064723|SNSTETLSPAKSPSSSTGSIASSR|4xPhospho [S8; S12; S14; S15];1xTMTpro [K11];1xTMTpro [N−Term]
**294**	ENSRNOP00000017156|SSDCDSPDSESDTGKK|1xCarbamidomethyl [C4];2xTMTpro [K15; K16];1xTMTpro [N−Term];2xPhospho [S9(99.8); S11(99.9)]
**295**	ENSRNOP00000034166|AMGDEDSDESGGGSPKPSPK|2xTMTpro [K16; K20];1xTMTpro [N−Term];2xPhospho [S7(100); S10(100)]
**296**	ENSRNOP00000052955|KETESEAEDDNLDDLER|1xTMTpro [K1];1xTMTpro [N−Term];1xPhospho [S5(100)]
**297**	ENSRNOP00000042235|DKGSPHSEGSDRYR|1xTMTpro [K2];1xTMTpro [N−Term];1xPhospho [S/Y]
**298**	ENSRNOP00000002429|RRSPSPYYSR.|1xTMTpro [N−Term];2xPhospho [S3(100); S5(97.4)]
**299**	ENSRNOP00000052955|TASPPPPPK|1xTMTpro [K9];1xTMTpro [N−Term];1xPhospho [S3(98.8)]
**300**	ENSRNOP00000017156|SSDCDSPDSESDTGKK|1xCarbamidomethyl [C4];2xTMTpro [K15; K16];1xTMTpro [N−Term];2xPhospho [S9(95.8); S/T]
**301**	ENSRNOP00000052955|KAQVSPQS|1xTMTpro [K1];1xTMTpro [N−Term];1xPhospho [S5(100)]
**302**	ENSRNOP00000044041|SSPAYCTSSSDITEPEQK|1xCarbamidomethyl [C6];1xTMTpro [K18];1xTMTpro [N−Term];3xPhospho [S8(95.3); S9(95.5); S10(95.5)]
**303**	ENSRNOP00000068479|GGGGNSSSSGSGSGSGSPSTGSSGSSSSPGARR|1xTMTpro [N−Term];1xPhospho [S/T]
**304**	ENSRNOP00000035734|EGSPLHK|1xTMTpro [K7];1xTMTpro [N−Term];1xPhospho [S3(100)]
**305**	ENSRNOP00000059423|KSHSSPSLNPDASPVTAK|2xTMTpro [K1; K18];1xTMTpro [N−Term];2xPhospho [S4(96.6); S13(100)]
**306**	ENSRNOP00000013600|VTKGETLT|1xTMTpro [K3];1xTMTpro [N−Term];1xPhospho [T8(100)]

**307**	ENSRNOP00000063671|SSPSKHQPPPIR|1xTMTpro [K5];1xTMTpro [N−Term];1xPhospho [S1(98.1)]
**308**	ENSRNOP00000044041|SSPAYCTSSSDITEPEQK|1xCarbamidomethyl [C6];1xTMTpro [K18];1xTMTpro [N−Term];3xPhospho [T7(95.5); S9(91.4); S10(91.4)]
**309**	ENSRNOP00000013071|RQSSSSAGDSDGEQEDSPAAR|1xTMTpro [N−Term];2xPhospho [S5(97.3); S]
**310**	ENSRNOP00000059915|GHTSPCGMK|1xCarbamidomethyl [C6];1xOxidation [M8];1xTMTpro [K9];1xTMTpro [N−Term];1xPhospho [T3(98.6)]
**311**	ENSRNOP00000025232|VHHDSPDPSPPR|1xTMTpro [N−Term];2xPhospho [S5(100); S9(100)]
**312**	ENSRNOP00000069092|MQFSFEGPEK|1xTMTpro [K10];1xTMTpro [N−Term];1xPhospho [S4(100)]
**313**	ENSRNOP00000052955|RASPSPPPK|1xTMTpro [K9];1xTMTpro [N−Term];2xPhospho [S3(100); S5(100)]
**314**	ENSRNOP00000072307|SHSGSSPEVDSK|1xTMTpro [K12];1xTMTpro [N−Term];2xPhospho [S5(99.9); S6(99.9)]
**315**	ENSRNOP00000063739|GGPAEEPSPRGSPR|1xTMTpro [N−Term];1xPhospho [S12(100)]
**316**	ENSRNOP00000054317|ETISAIDTSPKENTPVR|1xTMTpro [K11];1xTMTpro [N−Term];2xPhospho [S9(99.9); T14(100)]
**317**	ENSRNOP00000042392|SGYSSPGSPGTPGSRSR|1xTMTpro [N−Term];1xPhospho [S8(93.4)]
**318**	ENSRNOP00000057494|KASSSDDEGGPR|1xTMTpro [K1];1xTMTpro [N−Term];2xPhospho [S4(99.9); S5(100)]
**319**	ENSRNOP00000033816|DGPGDDRHSTKSDLPVHTR|1xTMTpro [K11];1xTMTpro [N−Term];2xPhospho [S12(97); S/T]
**320**	ENSRNOP00000052955|RYSPPIQR|1xTMTpro [N−Term];1xPhospho [S3(100)]
**321**	ENSRNOP00000069761|KFSSPPPLAVSR|1xTMTpro [K1];1xTMTpro [N−Term];1xPhospho [S4(95)]
**322**	ENSRNOP00000001334|VGECSGPAAVNNSSDTESVPSPR|1xCarbamidomethyl [C4];1xTMTpro [N−Term];1xPhospho [S13(92.9)]
**323**	ENSRNOP00000022351|AMGDEDSGDSDTSPKPSPK|2xTMTpro [K15; K19];1xTMTpro [N−Term];2xPhospho [S/T]
**324**	ENSRNOP00000052955|RVSHSPPPKQR|1xTMTpro [K9];1xTMTpro [N−Term];2xPhospho [S3(100); S5(100)]
**325**	ENSRNOP00000059423|SHSSPSLNPDASPVTAK|1xTMTpro [K17];1xTMTpro [N−Term];3xPhospho [S3(96.5); S4(99.7); S12(100)]
**326**	ENSRNOP00000022351|AIEKGCSQDDECVSLR|2xCarbamidomethyl [C6; C12];1xTMTpro [K4];1xTMTpro [N−Term];1xPhospho [S7(100)]
**327**	ENSRNOP00000022351|AMGDEDSGDSDTSPKPSPK|2xTMTpro [K15; K19];1xTMTpro [N−Term];2xPhospho [S7(100); S10(97.6)]
**328**	ENSRNOP00000011604|SPASVKSPGEAK|2xTMTpro [K6; K12];1xTMTpro [N−Term];1xPhospho [S4(100)]

**329**	ENSRNOP00000026539|MKGETPVNSTMSIGQAR|1xTMTpro [K2];1xTMTpro [N−Term];1xPhospho [T5(100)]
**330**	ENSRNOP00000036882|YGPVSVADTTGSGAADAKDDDDIDLFGSDDEEESEDAK|2xTMTpro [K18; K38];1xTMTpro [N−Term];1xPhospho [S28(95.7)]
**331**	ENSRNOP00000002429|SGSAHGSGK|1xTMTpro [K9];1xTMTpro [N−Term];1xPhospho [S7(100)]
**332**	ENSRNOP00000052955|RRTPSPPPR|1xTMTpro [N−Term];2xPhospho [T3(100); S5(100)]
**333**	ENSRNOP00000068926|KSSEGQEQDGGR|1xTMTpro [K1];1xTMTpro [N−Term];1xPhospho [S]
**334**	ENSRNOP00000068006|ASGQAFELILSPR|1xTMTpro [N−Term];1xPhospho [S11(100)]
**335**	ENSRNOP00000028636|VEHTSQGAK|1xTMTpro [K9];1xTMTpro [N−Term];1xPhospho [S5(100)]
**336**	ENSRNOP00000075032|DDGSWEVIEGYR|1xTMTpro [N−Term];1xPhospho [S4(100)]
**337**	ENSRNOP00000052955|AASPSPQSVR|1xTMTpro [N−Term];2xPhospho [S3(100); S5(100)]
**338**	ENSRNOP00000015073|YQQSSPSRLPR|1xTMTpro [N−Term];1xPhospho [S5(98.2)]
**339**	ENSRNOP00000052955|RLSPSASPPR|1xTMTpro [N−Term];2xPhospho [S3(100); S5(97.8)]
**340**	ENSRNOP00000071420|RLPSSPASPSPKGTPEK|2xTMTpro [K12; K17];1xTMTpro [N−Term];2xPhospho [S8(99.5); T14(100)]
**341**	ENSRNOP00000072307|QRSHSGSSPEVDSK|1xTMTpro [K14];1xTMTpro [N−Term];2xPhospho [S3(100); S5(100)]
**342**	ENSRNOP00000027344|VPLDGAFWIPRPPAGSPK|1xTMTpro [K18];1xTMTpro [N−Term];1xPhospho [S16(100)]
**343**	ENSRNOP00000044041|SSPAYCTSSSDITEPEQK|1xCarbamidomethyl [C6];1xTMTpro [K18];1xTMTpro [N−Term];2xPhospho [S8(95.5); S9(50)]
**344**	ENSRNOP00000075309|AQTNDSDSDTESKR|1xTMTpro [K13];1xTMTpro [N−Term];2xPhospho [S6(100); S8(100)]
**345**	ENSRNOP00000019696|KVSDGSEDEDEEEDEEESSEDSEDEEPPPKR|2xTMTpro [K1; K30];1xTMTpro [N−Term];3xPhospho [S6(100); S18(96.7); S]
**346**	ENSRNOP00000072307|RSLSGSSPCPK|1xCarbamidomethyl [C9];1xTMTpro [K11];1xTMTpro [N−Term];2xPhospho [S2(100); S4(100)]
**347**	ENSRNOP00000022120|SQSEEQSEASSEQLEQYAQSAEK|1xTMTpro [K23];1xTMTpro [N−Term];1xPhospho [S/Y]
